# Independent assessment and improvement of wheat genome sequence assemblies using Fosill jumping libraries

**DOI:** 10.1093/gigascience/giy053

**Published:** 2018-05-11

**Authors:** Fu-Hao Lu, Neil McKenzie, George Kettleborough, Darren Heavens, Matthew D Clark, Michael W Bevan

**Affiliations:** 1 John Innes Centre, Norwich Research Park, Norwich NR4 7UH, UK; 2 The Earlham Institute, Norwich Research Park, Norwich NR4 7UZ, UK

**Keywords:** wheat genome, genomics, assembly methods, Fosills, long-range genome assembly, Illumina, PacBio

## Abstract

**Background:**

The accurate sequencing and assembly of very large, often polyploid, genomes remains a challenging task, limiting long-range sequence information and phased sequence variation for applications such as plant breeding. The 15-Gb hexaploid bread wheat (*Triticum aestivum*) genome has been particularly challenging to sequence, and several different approaches have recently generated long-range assemblies. Mapping and understanding the types of assembly errors are important for optimising future sequencing and assembly approaches and for comparative genomics.

**Results:**

Here we use a Fosill 38-kb jumping library to assess medium and longer–range order of different publicly available wheat genome assemblies. Modifications to the Fosill protocol generated longer Illumina sequences and enabled comprehensive genome coverage. Analyses of two independent Bacterial Artificial Chromosome (BAC)-based chromosome-scale assemblies, two independent Illumina whole genome shotgun assemblies, and a hybrid Single Molecule Real Time (SMRT-PacBio) and short read (Illumina) assembly were carried out. We revealed a surprising scale and variety of discrepancies using Fosill mate-pair mapping and validated several of each class. In addition, Fosill mate-pairs were used to scaffold a whole genome Illumina assembly, leading to a 3-fold increase in N50 values.

**Conclusions:**

Our analyses, using an independent means to validate different wheat genome assemblies, show that whole genome shotgun assemblies based solely on Illumina sequences are significantly more accurate by all measures compared to BAC-based chromosome-scale assemblies and hybrid SMRT-Illumina approaches. Although current whole genome assemblies are reasonably accurate and useful, additional improvements will be needed to generate complete assemblies of wheat genomes using open-source, computationally efficient, and cost-effective methods.

## Background

Genome sequence assemblies are key foundations for many biological studies; therefore, the accuracy of sequence assemblies is a fundamental prerequisite for their use. Multiple types of differences in the information content of DNA molecules, from single nucleotide polymorphisms to large-scale structural variation, form part of natural genetic variation that can cause phenotypic variation [1, 2]. Distinguishing such *bona fide* variation from apparent variation generated by sequence and assembly methods is therefore a critically important activity in genomics.

Sequence assemblies are generally incomplete and contain multiple types of errors, reducing their information content. Gaps in assemblies can occur where no sequence reads were generated for that region, but this is now increasingly unlikely given the very deep coverage achievable by short read sequencing, improved sequence chemistry, and template preparation methods that avoid bias, such as that introduced by PCR [3]. Closely related repetitive DNA sequences can lead to incorrect joins in assemblies or to an unresolvable assembly graph that breaks an assembly. Assemblies can be either joined or broken inadvertently by closely related or polymorphic sequences that cause alternate, multiple, or collapsed assemblies, for example in assemblies of polyploid genomes [4]. Errors and incompleteness can also obscure important genomic information such as the correct order (phasing) of sequence variants.

A broad spectrum of sequence and assembly artefacts can be distinguished from natural sequence variation, structural variants identified, and sequence variation phased, using long-range sequence information. Sequences of long single molecules generated by PacBio Single Molecule Real Time (SMRT) and Nanopore technologies are increasingly used for *de novo* genome assembly [5, 6]. Linked read technologies such as 10xGenomics reads also have great promise for long-range ordering of scaffolds assembled from short reads, for defining extended haplotypes, and for identifying structural variation [7]. Chromatin interaction frequency data are also used to position sequence scaffolds into larger chromosome-scale pseudomolecular assemblies [8], while optical and genetic maps provide complementary ways of assessing chromosome-scale pseudomolecule assemblies [9]. Nevertheless, assessing sequence assemblies across smaller scales (100–1,000 kb) remains an essential task, as such local assembly errors can be propagated into larger pseudomolecule-scale alignments.

These improvements in sequencing and assembly are enabling the creation of genomic resources for very large and complex genomes, including those of grasses and gymnosperm trees, which have massive repetitive DNA tracts comprising about 80% of their genomes. The 22-Gb genome of loblolly pine (*Pinus taeda*), initially assembled from Illumina paired end sequence reads [10], has been significantly improved using SMRT sequencing [11]. 10xGenomics linked reads were used to generate an 8-fold increase in scaffold NG50 sizes of sugar pine (*P. lambertiana*) genome assemblies to nearly 2 Mb [12]. Bread wheat (*Triticum aestivum*) has a large 15-Gb allohexaploid genome comprising three closely related and separately maintained A, B, and D genomes [13]. Bacterial Artificial Chromosome (BAC)-based physical maps of flow-sorted chromosomes have been made to avoid misassembly of the separate genomes [14]. A 790-Mb tiling path of BACs from chromosome 3B has been sequenced and assembled [15], and represents the most complete available chromosome-scale assembly of wheat to date. Whole genome shotgun (WGS) [16] purified chromosome sequencing [17] approaches to wheat genome assembly have also been taken, but these assemblies were very fragmentary and incomplete. Recently a new approach to template preparation and assembly generated separate assemblies of the A, B, and D genomes, but although more complete, these remain quite fragmentary [18]. A near-complete and highly contiguous assembly of Illumina paired-end and mate-pair reads from wild emmer wheat (WEW, *Triticum turgidum ssp dicoccoides*), a tetraploid progenitor of bread wheat, has also recently been published [19], suggesting this approach also has promise for sequencing hexaploid wheat genomes. Finally, long SMRT sequence reads integrated with Illumina sequence coverage increased the size and contiguity of maize (*Zea mays*) [20], a diploid wheat progenitor [21], and hexaploid wheat genome assemblies [22]. It is therefore timely to assess the extent of different assembly error types in each of these different tetraploid and hexaploid wheat assemblies.

Generating accurate genome assemblies is essential for identifying haplotypes selected by breeders and for mapping large-scale structural variation contributing to agronomic performance [23]. Therefore, assessing the fidelity of wheat sequence assemblies generated by different strategies using a common approach is important for both determining optimal sequencing and assembly strategies and for identifying structural variation. Here we use very long, 40-kb mate-paired sequences of wheat fosmid clones to assess three different wheat whole genome assemblies and two BAC-based wheat chromosome assemblies. Our analyses have identified a range of error types in all assemblies and identify more optimal approaches to wheat genome assembly. We also integrated fosmid end-sequences into whole genome assembly scaffolds and substantially increased scaffold sizes of both fragmentary and more contiguous assemblies.

## Results

### Creating and assessing a wheat fosmid clone library

Fosmid clone libraries have been used to assess genome assemblies and identify structural variation in human [24, 25] and pine genomes [11]. Fosmids are used because DNA is cloned in a precise range of 38 ± 3 kb by efficient packaging in phage lambda and cohesive end circularisation. Fosmid clone inserts have been converted to Illumina sequencing templates to generate 38-Kb mate-pair “jumping libraries” and used to improve assemblies of the mouse genome [26]. In genomes with extensive tracts of very similar repeats and with closely-related homoeologous chromosomes that have been challenging to assemble, fosmid jumping libraries could provide an independent means to assess the fidelity of different wheat genome assemblies and to improve them. In particular, precisely spaced 38-kb paired sequences can identify a range of local assembly errors over scales that include current contig and scaffold sizes of wheat BAC and WGS assemblies.

To explore the potential of fosmid jumping libraries for assessing and improving different tetraploid and hexaploid wheat sequence assemblies, we first carried out a simulation of WGS assembly of three long 3.5- to 4.1-Mb scaffolds of wheat chromosome 3B generated by sequencing and assembling a manually curated physical map of BACs [15]. Simulation settings used different paired-end distances, read lengths, and sequence coverage on faux reads from chromosome 3B to assess how read-depth and read-length of 38-Kb mate-paired reads contributed to re-assembly of wheat scaffolds (Additional File 1). Addition of 38-Kb mate-pair reads was required for accurate and complete reconstruction of all three scaffolds under simulation conditions. Paired-end read lengths between 100 and 250 bp were then assessed using a common combination of mate-pair distances and sequence coverage. Reads of >200 bp were required for consistent re-assembly of all three scaffolds. Finally, simulation of sequence coverage of 38-kb mate-pair reads 250 bp long showed that consistent re-assembly of all three scaffolds required sequence coverage of at least 0.75x (Additional File 1). Taken together, these simulations showed that 38-Kb , paired-end, 250-bp reads with a sequence coverage of approximately 0.75x (>50x physical coverage, Additional File 2) could be used to guide and assess assemblies of the wheat genome.

The Fosill vector system was developed for converting fosmid clones to Illumina paired-end read templates [27]. We modified this Fosill conversion protocol to generate long paired-end, 250-bp Illumina reads to maximise library complexity and to minimise clonal- and PCR-based amplification bias. Both of these modifications were required to maximise unique matches of paired-end reads to the highly repetitive polyploid wheat genome and to maximise sequence coverage of the large genome. Additional File 2 describes modified protocols for library preparation and paired-end read analyses. These involved increasing the time of nick-translation to between 50-60 minutes on ice to generate inverse PCR products with a peak size distribution of 785–860 bp (Additional File 2). This minimised overlap of 250-bp reads from either end of the PCR product. For each pool of 5–10 M Fosill clones, a small sample of the circularised template was amplified for up to 16 cycles, and the minimum number of cycles required (generally 12–13) to generate sufficient template for sequencing was estimated.

**Figure 1:**
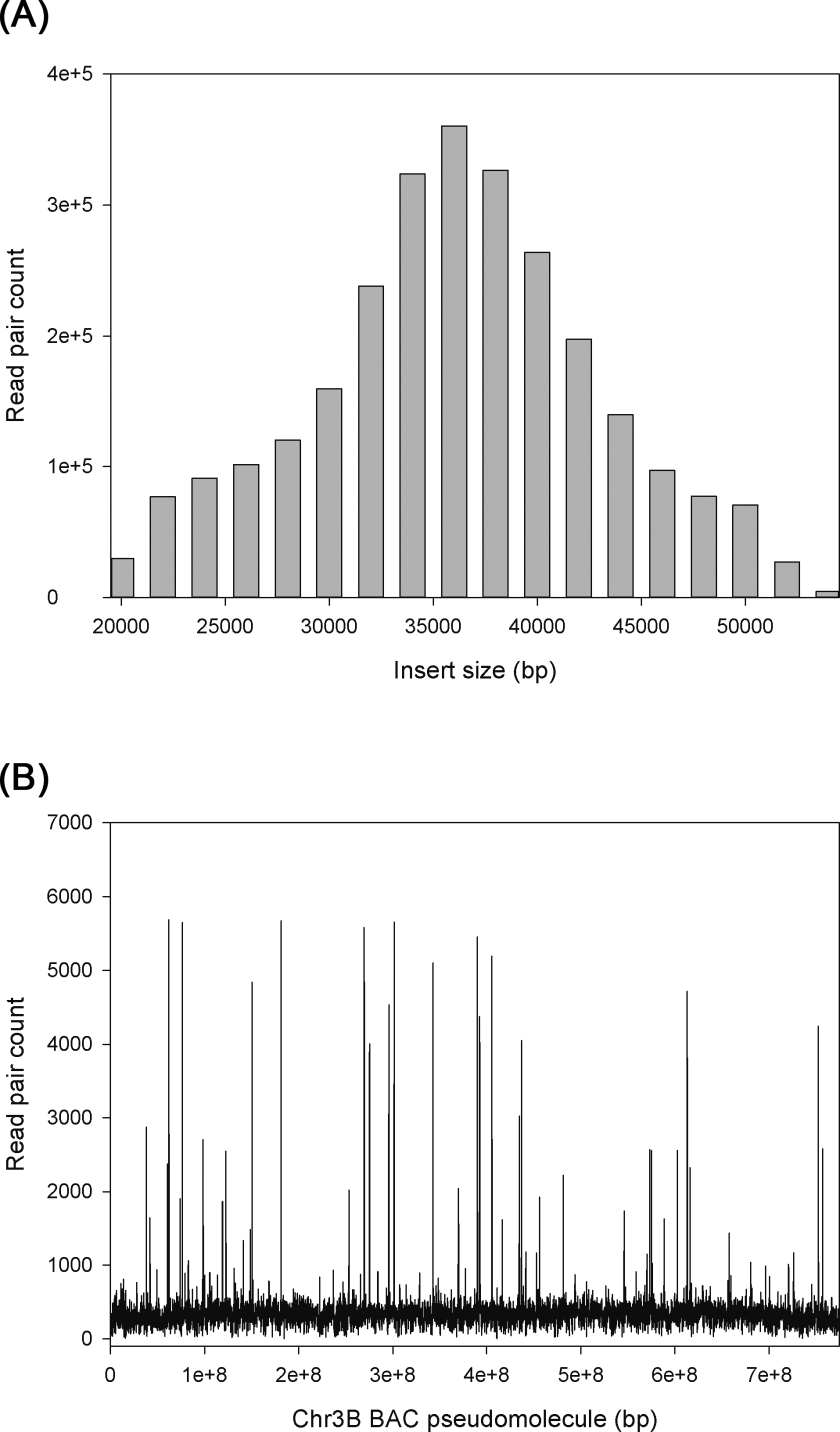
Determination of Fosill mate-pair distance distributions on chromosome 3B. **A)** 576-M, quality-controlled, paired-end sequences were mapped to the chromosome 3B pseudomolecule. A total 588,268 read pairs were mapped and the insert sizes calculated. The mean insert size was 37,725 Kb. **B)** Fosill mate-pairs were mapped in 100-kb bins along chromosome 3B to assess the depth and evenness of coverage. Coverage was generally even across the entire chromosome, with approximately 30 very high copy peaks that are probably due to Fosill mate-pairs from highly related 40-kb+ regions from across the genome. Most mate-pairs mapped to a depth of <=5 and were used for subsequent analyses.

Table 1 in Additional File 2 summarises the Fosill libraries produced and the paired-end sequences generated from them. Paired-end reads that overlapped each other on the template were discarded (2.61%), while 11.91% of the raw reads were excluded after vector/adapter sequence and quality trimming. The final number of 576-M paired-end sequences (85.5% of the total reads) were generated from 54.61-M Fosill clones (1.8x sequence coverage, 138x physical coverage). These were then mapped to the chromosome 3B pseudomolecule to measure the insert size distribution of the libraries (Additional File 3). Fig. 1A shows the size distribution of 588,268 mapped read pairs, which had a mean estimated insert size of approximately 37,725 bp. This is the expected insert size range in the Fosill4 vector [27] and demonstrated successful size selection during packaging. Fig. 1B shows the distribution of mate-pairs mapped in 100-kb windows across chromosome 3B BAC pseudomolecule. Reads with a depth of ″5 covered 494 Mb of the total 833-Mb chromosome, accounting for 59% of the chromosome sequence. Their even distribution across the pseudomolecule indicated that the libraries were representative of the entire chromosome. There were approximately 30 distinct peaks of greatly increased read-depth (Fig. 1B) in the 100-kb windows across chromosome 3B. These probably correspond to mate-pairs spanning approximately 40-Kb repeated regions common to multiple genomic loci. These reads account for 80% of the alignments but covered only 4.3% of chromosome 3B. For all subsequent analyses, only Fosill mate-pairs of sequence depth <=5 were used. Finally, reads that mapped to multiple locations, which lacked a paired read in the expected genomic location or which had a paired read in the incorrect orientation, were removed.

**Table 1:** The consistency of mapping is shown according to the number of assemblies with consistent matches, and the percentage of bases included in consistent matches to Fosill mate-pairs

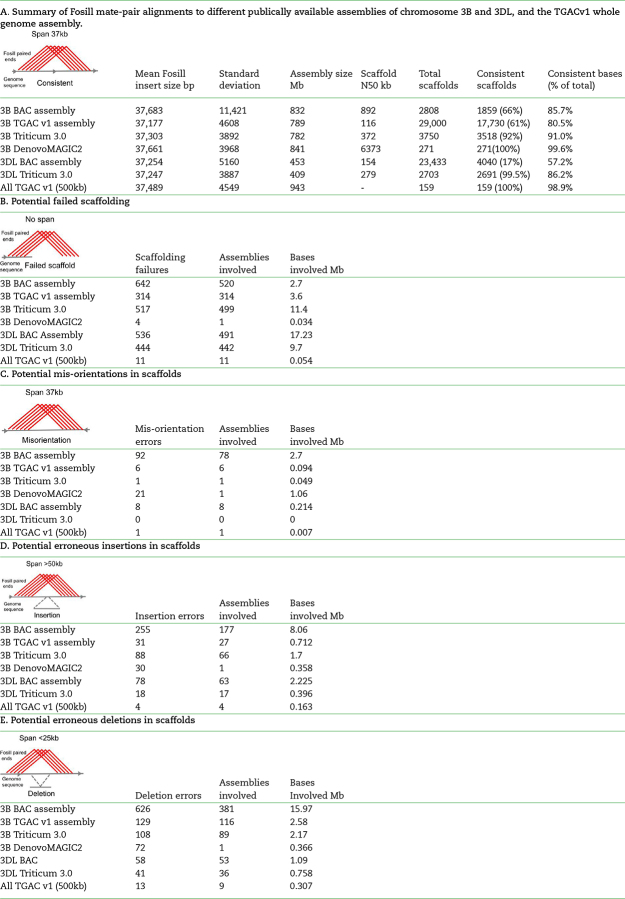

### Using Fosill mate pairs to assess wheat chromosome sequence assemblies

**Figure 2:**
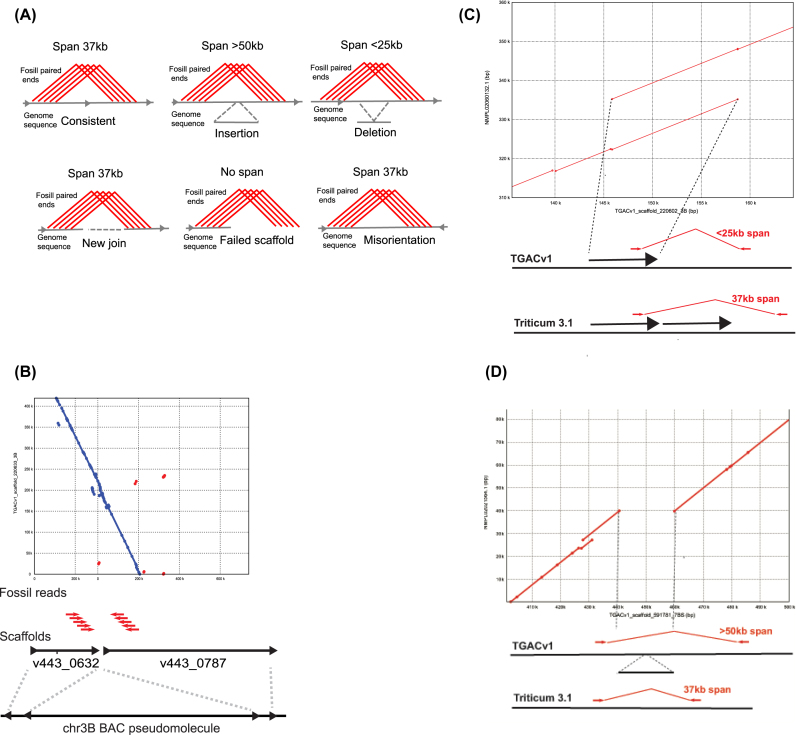
Using Fosill mate-pair matches to identify discrepancies in wheat chromosome and genome assemblies. **A**) The schematic describes different classes of matches of Fosill mate-pair sequences to wheat chromosome and genome assemblies. Consistent assemblies matched a span of ≥5 mate-pairs in a sliding 10-kb “driver” window that matched their mate in a 20-kb “follower” window at a distance of 37 kb +/- sd in the correct orientation. Where mate-pairs spanned more than 50 kb (approximately 3 sd), this was construed to be due to an aberrant insertion in the underlying assembly. Spans <25 kb (approximately 3 sd) were construed to be due to an aberrant deletion in the assembly. Mis-orientations of the mate-pairs indicated a mis-oriented assembly, and no span a mis-join in the assembly. New joins were also identified. Drawing not to scale. **B**) An example of a mis-join of the BAC-based assembly of chromosome 3B. Two scaffolds, v443_0362 and v443_0787, were originally assembled at opposite ends of chromosome 3B 730 Mb apart. Matches to Fosills indicated that these two scaffolds could be re-assembled together with v443_0362 in the opposite orientation. The Mummer plot shows that this join is supported by TGACv1 scaffold_220633_3B. Drawing not to scale. **C**) An example of an aberrant deletion in TGACv1 scaffold 220602 on chromosome 3B. Assembly missed a duplicate copy of a 12-Kb repeat (represented by an arrow) that was identified as a discrepancy in Fosill mate-pair matches. Comparison to a Triticum 3.0 scaffold identifies the predicted missing copy of the repeat. Drawing not to scale. **D**) An example of an aberrant insertion in TGAv1 scaffold 591,781 on chromosome 7BS detected by Fosill mate-pair matches of >50 Kb. Comparison to the Triticum 3.0 assembly of the same regions identifies the mis-assembled insertion. Drawing not to scale.

The even representation of long mate-paired reads across the chromosome 3B pseudomolecule indicated their suitability for assessing wheat sequence assemblies and for making new joins in wheat sequence scaffolds. For assessing assemblies, a windows-based filter was developed to identify sets of ≥5 unique neighbouring Fosill sequence reads in a “driver” window of <10 kb and their ≥5 mate-pair reads in a “follower” window of <20 kb on chromosome and genome assemblies. The vast proportion of mate-paired reads fell within this distance distribution (Additional File 3, Fig. 1). Using this approach to map Fosill reads, we aimed to identify different types of paired-end matches to genome sequence assemblies. These can be used to identify genome assemblies consistent with the 37.7-Kb mate-pair distances +/- sd, to identify possible new joins between assemblies, and to identify different types of inconsistencies in the range of current publicly available wheat genome assemblies. Fig. 2A illustrates the possible types of Fosill paired-end matches to assemblies.

Table 1A–E show the outcomes of mapping Fosill paired-end reads to BAC-based bread wheat chromosome assemblies of chromosome 3B [15], TGACv1 Illumina assemblies of 3B [18], the Triticum 3.0 whole genome assembly of Pacbio SMRT,and Illumina sequences of chromosomes 3B and 3DL (Additional Files 4 and 5) [22], and DeNovoMagic assemblies of Illumina sequences from WEW chromosome 3B [19]. We also assessed an assembly of hexaploid wheat chromosome 3DL from sequenced BACs in a minimal tiling path using an automated pipeline (Additional File 6). Finally, a set of larger whole genome assemblies of the TGACv1 Illumina wheat genome was also assessed. These assemblies represent diverse approaches to sequencing wheat chromosomes and chromosome arms, including manually curated and automated BAC-based assemblies, two different Illumina-based assembly methods, and a combined Illumina and Pacific Biosciences SMRT assembly of wheat chromosomes.

Variation in Fosill insert sizes were consistent across the TGACv1, Triticum 3.0, and DeNovo Magic whole genome assemblies of chromosome 3B and the 3DL BAC assemblies. In contrast, chromosome 3B BAC assemblies had a higher variation of insert sizes (Table 1A). This may be due to a higher proportion of mis-assemblies in the 3B BAC assembly that could have introduced or removed small tracts of sequences, and possibly due to the use of a mixture of 454 and Illumina sequences in the assembly. This variation in Fosill mate-pair matches did not contribute to assessment of assembly accuracy. The accuracy of assemblies was estimated by counting the bases included in correctly sized windows (mean insert size +/-sd) of Fosill mate-pair reads and by the proportion of assemblies/scaffolds that were fully consistent with Fosill mate-pair windows along their length. The un-edited BAC-based scaffolds of chromosome 3DL were the least accurate, with only 17% of the assemblies covered with consistent fossil mate-pair matches, and 57.2% of the sequence included under consistent mate-pair matches (Table 1A). The 3B BAC assemblies, which were extensively manually edited, were considerably more accurate, with 66% consistent assemblies and 85.7% of sequences in consistent windows. Looking at the TGACv1 3B assemblies, 61% of scaffolds were consistent and 80.5% of sequences were contained within consistent Fosill windows. In contrast, larger TGACv1 assemblies from the whole genome were all consistent with mate-pair windows, and 98.9% of the sequences were in consistent windows. These differences between TGACv1 3B assemblies are most likely due to the inclusion of many shorter assemblies in the 3B assembly that limit the potential for 38-Kb mate-pair mapping; for example, there will be a low proportion of matches at the ends of assemblies. The Triticum 3.0 WGS assembly of 3B had 92% consistent assemblies and 91% of sequences within consistent Fosill windows. Similarly, the Triticum 3.0 WGS assembly of chromosome 3DL had 99.5% assemblies and 86.2% of sequences in consistent windows. The DeNovo Magic WGS assembly of *T. turgidum* 3B contained 99.6% of sequences in consistent Fosill windows. As these assemblies were integrated into a single pseudomolecule, the measure of the number of correct scaffolds was 100%.

Four different classes of discrepancies that may be due to assembly problems were assessed using Fosill mate pair mapping to assemblies: failed scaffolding, in which scaffolds had matches to only one end of Fosill end-sequences and which may need to be broken; orientation errors, in which the direction of one region of a scaffold is consistently reversed with respect to flanking regions; insertions, in which the span of Fosill mate-pair matches is greater than expected; and deletions, in which mate-pair spans are less than expected. These results are summarised in Table 1B–E. Of these potential error types, the most frequent were the potential erroneous joining of assemblies. These were highest in the BAC assemblies of hexaploid wheat and lowest in the DenovoMAGIC assembly of WEW 3B. An example of this is shown in Fig. 1B, where two BAC-based scaffolds were assembled at either end of chromosome 3B. Fosill mapping evidence, supported by TGACv1 assemblies, showed that the two scaffolds can be merged in opposite orientation to that originally assembled. Fig. 2C reveals a 12-Kb deletion in a TGACv1 assembly that was due to a missing tandem duplication of the repeat, as validated by comparison with the Triticum 3.0 assembly. An aberrant insertion in a TGACv1 scaffold identified by Fosill mate-pair mapping was also validated by comparison with the Triticum 3.0 assembly (Fig. 2D).

The TGACv1 large assemblies have relatively low numbers of mis-assemblies. The Triticum 3.0 assemblies of both 3B and 3DL had a consistently large number of potential mis-assemblies, with about 400–500 per chromosome or chromosome arm, affecting about 10 Mb of sequence region. Potential deletion errors, in which assemblies may be missing sequences, were most frequent in the BAC assembly of chromosome 3B and were also the most frequent type of error in the DenovoMAGIC assembly. Deletions were least frequent in the TGACv1 whole genome assembly. Potential erroneous insertions were less frequent than deletions, with the highest rates of both types of potential error in BAC-based assemblies. In general, potentially erroneous deletions were more common in all assemblies than insertions. Mis-orientations were the rarest potential error type, being most prevalent in manual assembled 3B BAC scaffolds, essentially absent from TGACv1 and Triticum 3.0 assemblies, but more frequent in the DeNovo Magic WEW 3B assembly.

### Using Fosill mate-pairs to create more contiguous assemblies

**Figure 3:**
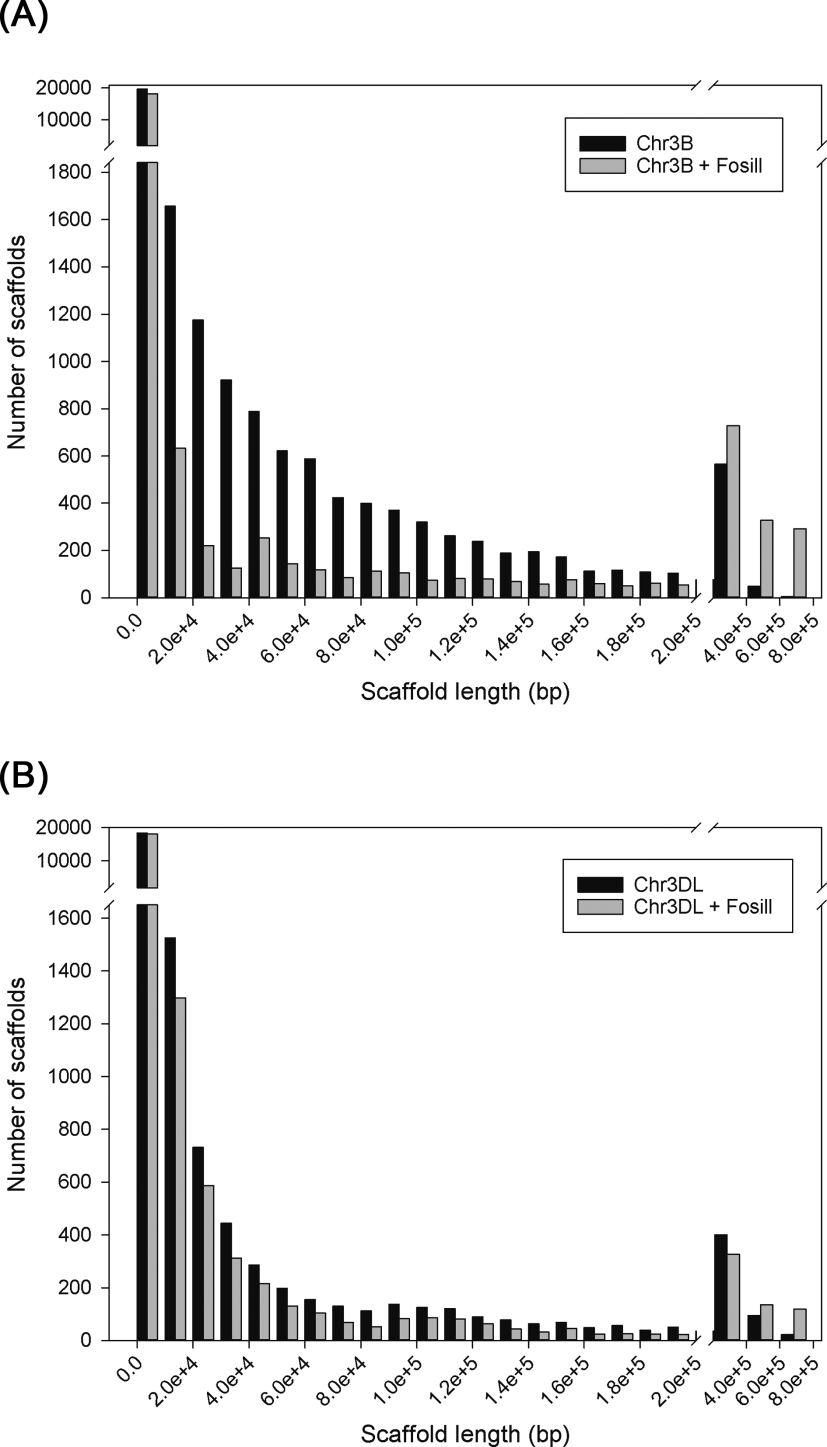
Increasing assembly contiguity using Fosill matches. **A**) Fosill mate-pair reads were used to link scaffolds of TGACv1 Illumina assemblies from chromosome 3B. The distribution of scaffold lengths and the number of scaffolds in each size range are shown before (dark bars) and after (grey bars) Fosill scaffolding. The numbers of smaller scaffolds are reduced and the numbers of larger scaffolds are increased by Fosill scaffolding, showing successful further assembly. **B**) Fosill mate-pair reads were used to link scaffolds of BAC-based assemblies of chromosome 3DL. The distribution of scaffold lengths and the number of scaffolds in each size range is shown before (dark bars) and after (grey bars) Fosill scaffolding. The numbers of smaller scaffolds are reduced and the numbers of larger scaffolds are increased by Fosill scaffolding, showing successful further assembly.

The wheat Fosill library was also used to create new joins in different assemblies. Table 2A shows that Fosill mate-pair reads made 267 new links between 477 chromosome 3B BAC scaffolds. Where available, TGACv1 3B assemblies spanning the new links precisely (124 cases), supporting the new join, and no examples were found where the new Fosill joins linked the wrong neighbours or the wrong strand. We then applied the Fosill mate pairs to make new joins in chromosome 3B TGACv1 assemblies and chromosome 3DL BAC assemblies. Table 2B shows the total assembly sizes were increased, while the number of scaffolds in the assemblies was decreased and the scaffold n10 more than doubled in size. This showed, as predicted by simulations (Fig. 1), that 38-Kb mate-pair reads can make new links that substantially improve contiguity of both WGS and BAC-based assemblies. Where available, independent assemblies supported these new Fosill-based links. Fig. 3 shows the distribution of scaffold sizes and numbers before and after Fosill linking on TGACv1 chromosome 3B (panel A) and chromosome 3DL BAC (panel B) assemblies. Increases in the numbers of larger assemblies and concomitant reduction in the numbers of smaller assemblies after Fosill joining was more apparent in the chromosome 3B WGS scaffolds than in the 3DL BAC scaffolds. This may reflect the fewer joins needed in the less fragmentary 3B assembly (2,808 scaffolds) than the very fragmented 3DL assembly (23,433 scaffolds).

**Table 2:** Using Fosill paired-end sequences to improve wheat chromosome assemblies.

2A Summary of new links made between BAC scaffolds on chromosome 3B		
267 new links	Strand	Validated by TGACv1
37 links ≤40 kb on pseudomolecule	20 correct strands	12
	17 reverse strands	7
147 links >40 kb on pseudomolecule	73 correct strands	31
	74 reverse strands	38
83 links between scaffolds not assigned to the pseudomolecule	-	36

2B. Summary of changes in assemblies of chromosome 3B TGACv1 and chromosome 3DL BAC assemblies				
	chr3B TGACv1		chr3DL BACs	
	Before	After	Before	After
Bases (bp)	789,970,040	846,817,359	452,947,627	463,673,958
Assemblies	29,090	22,014	23,433	21,985
Number scaffolds >n50	2,020	644	790	721
Min	500	500	501	501
n10	293,318	947,439	463,110	933,142
n50	116,546	398,569	154,985	286,993
Max	739,616	2,867,878	1,240.092	1,942,124

**Figure 4:**
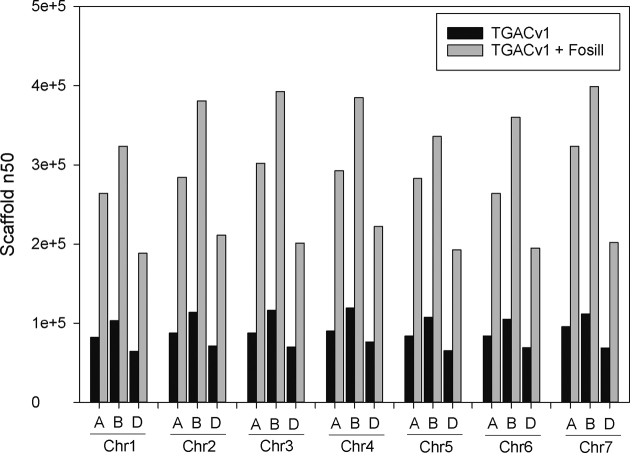
Fosill-mediated scaffolding of TGACv1 Illumina assemblies of the wheat genome. The 21 chromosomes are shown with their scaffold N50 values before (black bars) and after (grey bars) Fosill-mediated scaffolding.

Based on these improvements in both BAC-based and WGS scaffold contiguity by integrating Fosill mate-pair reads, we re-scaffolded the complete TGACv1 WGS assembly of the wheat variety Chinese Spring 42 [19]. Fig. 4 and Additional File 7 show the scaffold sizes of each chromosome arm before and after integration of Fosill mate-pairs in the new TGACv2 assembly. Substantial increases in scaffold N50 of between 2.7- and 3.2-fold were achieved. The largest scaffolds increased is size between 1.5- and 3.2-fold, with the largest scaffold of 2.8 Mb on chromosome 3B.

## Discussion

Bread wheat is one of the three major cereals that we depend on for our nutrition, and generating accurate long-range assemblies is essential for new genomics-led approaches to crop improvement. However, its genome has been exceptionally challenging to sequence due to its polyploid composition of three closely related large genomes and extensive tracts of very similar repetitive sequences. Two strategies have been followed to deal with this genomic complexity: the first used BAC clones made from purified chromosomal DNA to reduce the complexity of chromosome-specific assemblies [15]; the second set of approaches uses different types of WGS sequence technologies and assembly methods [18, 19, 22]. At this stage of wheat genome sequencing, when assemblies from these complementary and contending approaches are available for use, it is timely to assess the accuracy of these different assemblies. For this, we mapped precise 38-kb Fosill long mate-pair reads to measure errors in different assemblies of chromosome 3B and the long arm of chromosome 3DL. We also used these Fosill mate-pair reads to increase whole genome assembly contiguity.

In order to maximise the accuracy of Fosill mate-pair read mapping to the A, B, or D genomes and to repetitive regions of the hexaploid wheat genome, we modified the template conversion protocol of the Fosill 4 vector system [26] to generate longer paired 250-bp Illumina sequence reads. Nick-translation reactions to extend Nb.BbvCI nicks were optimised to generate an Illumina sequencing template between 750 and 1,000 bp. PCR amplification of re-circularised products was optimised to reduce amplification to the minimum required for efficient sequencing of a large library. Overall, 576.5 M read pairs were generated from 55.1 M clones (Additional File 2), generating 1.8x total sequence coverage of the wheat genome. When reads were mapped to chromosome 3B sequence assemblies, a consistent size distribution around 37.7 Kb was observed (Fig. 1A), demonstrating correct phage lambda packaging and processing. Read depth varied several thousand-fold along chromosome 3B, likely due to matches of read-pairs to highly repetitive regions from across the genome. Consequently, only read-pairs with depth ≤5 were used. Using this filter, we obtained sequence coverage of nearly 60% of the 833-Mb BAC-based chromosome 3B assembly. Simulations indicated that 0.75x sequence coverage of paired-end 250-bp reads was effective in creating long-range assemblies of wheat (Additional File 1); therefore we used Fosill read mapping for subsequent analyses.

Fosmid mate-pair sequences have been used to close gaps [28] and detect structural variation [24] in the human genome. Different types of variation, including insertions, deletions, and inversions spanning tens to hundreds of kb, were detected. Larger scale errors, for example, generated in the creation of pseudomolecules spanning chromosomes, are more readily detected using optical mapping and chromatin conformation methods [27]. Fosill reads were mapped to different assemblies of chromosome 3B and the long arm of chromosome 3D in order to compare the full range of current publicly available hexaploid and tetraploid wheat assemblies. Table 1A–E shows the types of inconsistencies detected in different wheat assemblies using this approach. Looking first at the proportion of bases in different assemblies that were fully consistent with mapped 38-Kb mate-pair reads (Table 1A), the DenovoMAGIC Illumina-based WEW assembly, the larger TGACv1 assemblies, and the merged SMRT long-read and Illumina short read Triticum 3.0 assembly of chromosome 3B had, respectively, 99.6%, 98.9%,and 91.0% of bases in consistent Fosill windows. The manually curated BAC-based assembly of 3B had 85.7% of consistent bases, while the TGACv1 3B assembly had 80.5% of assembled sequence in consistent windows. The difference between the total TGACv1 3B assembly probably reflects the inclusion of shorter assemblies in the TGACv1 3B assembly that are shorter than 38 Kb. The non-curated BAC assembly of chromosome 3DL was the least accurate according to this measure, with only 57.2% of bases in consistent windows. These data demonstrate the superior accuracy of *de novo* whole genome sequencing strategies that incorporate deep and long 250-bp Illumina paired-end and mate-pair sequence coverage and the relative accuracy of long-range assemblies generated by mate-pair assembly strategies [18, 19] compared to BAC-based and SMRT strategies [15, 22].

The most frequent type of inconsistency identified by Fosill mapping was the potential incorrect joining of assemblies (Table 1B). Illumina strategies using long mate-pair information produced the fewest incorrect joins, while BAC-based assemblies produced the most. Interestingly, the hybrid Triticum 3.0 assembly of both 3B and 3DL made from PacBio SMRT reads combined with 150-bp Illumina paired end reads [11] had more potential assembly errors than the Illumina-only assemblies, with 517 and 444 potential mis-assemblies on chromosomes 3B and 3DL, respectively. While the merged Triticum 3.0 assembly is more complete and contains no unknown bases, it was based on relatively short Illumina reads (150-bp paired end reads vs 250-bp paired end reads in the TGAC v1 and DenovoMAGIC assemblies) and did not include any longer Illumina mate-pair sequences. The multiple merging steps used in generating the Triticum 3.0 assembly may also contribute to the relatively high numbers of mis-assemblies. Assembly methods may also need further optimisation to utilize fully the potential of SMRT long reads. Furthermore, integrating long 250-bp Illumina reads into mega-reads may improve assemblies by distinguishing very closely related sequences, such as repeat regions from homoeologous chromosomes.

Potential deletion events were also quite common in all assemblies and were the most common inconsistencies detected in DenovoMAGIC assemblies of 3B. The sizes of these events are not known precisely, but they have a minimum size of 12 kb (Table 1E). These probably arise from missing tracts of near-identical sequence in assemblies. Similarly, potential insertions may arise from the incorrect integration of near-identical sequences into assemblies. The observation that potential deletions are more frequent than potential insertions suggests that all WGS-alone assembly strategies could achieve more complete assemblies of the wheat genome, such as that achieved using PacBio SMRT sequence assemblies. Finally, potential mis-orientations/inversions of assemblies are more common in the DevoMAGIC assembly of 3B than the other whole genome assemblies. Although this approach has yet to be fully described, mis-orientations may reflect more relaxed criteria for linking scaffolds than related Illumina-based assembly and scaffolding approaches [18].

How much more accurate can the best current assemblies of bread wheat and WEW be, judging by their assemblies of chromosome 3B? Fosmid end-mapping to 2005 versions of human genome assemblies [24] identified 297 longer range discrepancies in the 3.2-Gb genome. Scaling from chromosome 3B (0.8 Gb) with 127 potential inconsistencies, our analyses predict 480 discrepancies per 3 Gb of WEW genome assembly, roughly twice the error frequency of 2005 versions of the human genome. It is likely that a DenovoMAGIC version of the hexaploid bread wheat genome will achieve similar high levels of accuracy and coverage.

Three-fold increases in the scaffold N50 sizes of the TGACv1 whole genome assembly were achieved by an additional scaffolding step using Fosill mate-pairs. In addition to making a more useful genomic resource, this additional scaffolding shows the relatively fragmentary but highly accurate TGACv1 assembly has the potential for substantial further improvement as an open-source and computationally efficient approach to assembling multiple wheat genomes [18]. For example, the direct integration of linked read technologies [29] and Nanopore long reads [30] into this assembly process should substantially increase contiguity, and directly and precisely identify a wide range of structural and phased sequence variation in wheat genome assemblies that are required for trait analyses and for accelerating breeding.

## Methods

Detailed descriptions of experimental and computational procedures are shown in Additional Files. These describe simulations of 38-kb mate-pair reads for assembly (Additional File 1), production and sequencing of Fosill libraries (Additional File 2), mapping of Fosill mate-pairs (Additional File 3), and physical mapping and sequencing of BACs from chromosome 3DL (Additional File 6).

### General bioinformatics

All analytical pipelines have been deposited in GitHub and in SciCrunch (NGSimple, RRID:SCR_016165; ReadCleaner4Scaffolding, RRID:SCR_016166). Relevant links are shown in the manuscript and Additional Files. Joinable read pairs from Illumina Miseq or HiSeq sequencing were removed using FLASH v1.2.11 [31]. Ligation adaptors in reads were trimmed off using CutAdapt v1.6 [32]. Sequencing primer sequences and low-quality sequences in reads were removed using Trimmomatic v0.32 [33]. The resulting reads were evaluated using FastQC v1.2.11 [34].

Trimmed reads were further filtered using ReadCleaner4Scaffolding pipeline [35]. Both mates in each pair were mapped to chr3B BAC scaffolds using bowtie v1.0.1 (Bowtie, RRID:SCR_005476) [36]. And then the Picard MarkDuplicates v1.108 (Picard, RRID:SCR_006525) [37] was used to remove the duplicates as single reads. A read depth threshold was used to remove the repeat-like reads by plotting the summary of output from samtools depth, and the reads mapped to those regions with higher depth were not used for scaffolding. The remaining reads were subjected to removal again as pairs.

Those reads mapped to multiple positions, whose mates were not mapped or had the wrong orientation, were removed. A window size filter was applied to identify sets of ≥5 neighbouring reads in sliding windows of <10 kb that had all their mates in a following window of <20 kb. Variations of the expected distance between mate-pairs (average ± sd) of approximately 3 sd was used to identify potential assembly discrepancies.

## Data Availability

Fosill mate-pair sequence reads from *Triticum aestivum* Chinese Spring 42 generated in this study have been submitted to the EBI European Nucleotide Archive (ENA) and are available in study accession PRJEB23322. The TGAC v2 *Triticum aestivum* Chinese Spring 42 whole genome assembly is available in ENA study accession PRJEB23893. *Triticum aestivum* Chinese Spring 42 chromosome 3DL BAC scaffold sequences are available in ENA study accession PRJEB23358. Snapshots of the pipeline and data are available in the *GigaScience* GigaDB repository [38].

## Availability of supporting source code and requirements

Project names: NGSimple, & ReadCleaner4Scaffolding

Project home pages: https://github.com/lufuhao/NGSimple & https://github.com/lufuhao/ReadCleaner4Scaffolding

Operating system: Linux

Programming language: Perl, Bash

Other requirements: See bash scripts in ProgramRoot/utils/

License: GNU GPL v3

RRID: NGSimple, RRID:SCR_016165; ReadCleaner4Scaffolding, RRID:SCR_016166

## Additional Files

Additional File 1. Genome Assembly Simulation.doc

Additional File 2. Fosill Library Production.doc

Additional File 3. Fosill Mate-Pair Mapping.doc

Additional File 4 Triticum 3.1 PacBio assemblies of chromosome 3B.xls

Additional File 5. Triticum 3.1 PacBio assemblies of chromosome 3DL.xls

Additional File 6. Sequencing Chromosome 3DL BAC Minimal Tiling Path.doc

Additional File 7. TGACv2 chromosome scaffolds.xls

## Abbreviations

BAC: Bacterial Artificial Chromosome; BBSRC: Biological and Biotechnological Sciences Research Council; sd: standard deviation; SMRT: Single Molecule Real Time; WEW: wild emmer wheat; WGS: whole genome shotgun.

## Declarations

The authors declare they have no competing interests.

## Funding

This work was supported by a Biological and Biotechnological Sciences Research Council (BBSRC) strategic LOLA award to MWB (BB/J00328X/1 and MDC (BB/J003743/1), The FP7 Triticeae Genome Project to MWB, and a BBSRC Institute Strategic Programme Grant (GEN) BB/P013511/1 to MWB. BBSRC Institute Strategic Programme Grant (BB/J004669/1) and Core Strategic Programme Grant (BB/CSP17270/1) also supported work at the Earlham Institute. Sequencing was delivered via the BBSRC National Capability in Genomics (BB/J010375/1) at the Earlham Institute and performed by members of the Genomics Pipelines Group.

## Authors’ contributions

M.W.B. conceived and coordinated the project, and wrote the manuscript; F.-H.L. planned and carried out bioinformatics analyses; N.McK. constructed the Fosill libraries; G.K. and M.D.C. sequenced chromosome 3DL BACs and managed sequence data; and D.H. managed all sequencing library production and Illumina sequencing.

## Supplementary Material

GIGA-D-17-00308_Original_Submission.pdf

GIGA-D-17-00308_Revision_1.pdf

GIGA-D-17-00308_Revision_2.pdf

Response_to_Reviewer_Comments_Original_Submission.pdf

Response_to_Reviewer_Comments_Revision_1.pdf

Reviewer_1_Report_(Original_Submission) -- Martin Mascher 12/27/2017 Reviewed

Reviewer_2_Report_(Original_Submission) -- Sachiko Isobe 2/12/2018 Reviewed

Reviewer_2_Report_(Revision_1) -- Sachiko Isobe 4/9/2018 Reviewed

Supplemental material
