# Supplementary material for: Independent assessment and improvement of wheat genome sequence assemblies using Fosill jumping libraries
Source: Gigascience. 2018 May 11;7(5):giy053. doi: 10.1093/gigascience/giy053 (PMC5967450; doi:10.1093/gigascience/giy053)
Supplement: GIGA-D-17-00308_Revision_2.pdf [file giy053_giga-d-17-00308_revision_2.pdf]

GigaScience

Independent assessment and improvement of wheat genome sequence assemblies using Fosill jumping libraries.

--Manuscript Draft--

**Manuscript Number:**	GIGA-D-17-00308R2
**Full Title:**	Independent assessment and improvement of wheat genome sequence assemblies using Fosill jumping libraries.
**Article Type:**	Research
**Funding Information:**	Biotechnology and Biological Sciences Research Council (BB/J00328X/1) — Prof Michael Webster Bevan<br>Biotechnology and Biological Sciences Research Council (BB/J003743/1) — DR Matthew D Clark<br>Biotechnology and Biological Sciences Research Council (BB/P013511/1) — Prof Michael Webster Bevan<br>Seventh Framework Programme (Triticeae Genome) — Prof Michael Webster Bevan<br>Biotechnology and Biological Sciences Research Council (BB/CSP17270/1) — DR Matthew D Clark<br>Biotechnology and Biological Sciences Research Council (BB/J010375/1) — DR Matthew D Clark
**Abstract:**	Background<br>The accurate sequencing and assembly of very large, often polyploid, genomes remain a challenging task, limiting long-range sequence information and phased sequence variation for applications such as plant breeding. The 15 Gb hexaploid bread wheat genome has been particularly challenging to sequence, and several different approaches have recently generated long-range assemblies. Mapping and understanding the types of assembly errors is important for optimising future sequencing and assembly approaches and for comparative genomics.<br>Results<br>Here we use a Fosill 38 Kb jumping library to assess medium and longer-range order of different publicly available wheat genome assemblies. Modifications to the Fosill protocol generated longer Illumina sequences and enabled comprehensive genome coverage. Analyses of two independent BAC-based chromosome-scale assemblies, two independent Illumina whole genome shotgun assemblies, and a hybrid Single Molecule Real Time (SMRT-PacBio) and short read (Illumina) assembly were carried out. We revealed a surprising scale and variety of discrepancies using Fosill mate-pair mapping and validated several of each class. In addition, Fosill mate-pairs were used to scaffold a whole genome Illumina assembly, leading to a three-fold increase in N50 values.<br>Conclusions<br>Our analyses, using an independent means to validate different wheat genome assemblies, show that whole genome shotgun assemblies based solely on Illumina sequences are significantly more accurate by all measures compared to BAC-based chromosome-scale assemblies and hybrid SMRT-Illumina approaches. Although current whole genome assemblies are reasonably accurate and useful, additional improvements will be needed to generate complete assemblies of wheat genomes using open-source, computationally efficient and cost-effective methods.
**Corresponding Author:**	Michael Webster Bevan, PhD<br>John Innes Centre<br>Norwich, UNITED KINGDOM
**Corresponding Author Secondary Information:**	
**Corresponding Author's Institution:**	John Innes Centre

Powered by Editorial Manager® and ProduXion Manager® from Aries Systems Corporation

**Corresponding Author's Secondary Institution:**	
**First Author:**	Fu-Hao Lu, PhD
**First Author Secondary Information:**	
**Order of Authors:**	Fu-Hao Lu, PhD
	Neil McKenzie, BSc
	George Kettleborough, PhD
	Darren Heavens, BSc
	Matthew D Clark, PhD
	Michael Webster Bevan, PhD
**Order of Authors Secondary Information:**	
**Response to Reviewers:**	Responses to Reviewers' comments GIGA-D-17-00308<br><br>Reviewer x<br><br>This reviewer makes important points about the comparison of different types of assemblies and about how they should not be approached as "equivalent assemblies". This is important because different assembly types (BAC vs WGS) may have different error types at different scales; eg BAC-based assemblies are based on a minimal tiling path (MTP) of BACS (often supported by other data types such as genetic maps), and the longer- range scale assembly accuracy of such MTPs may be more accurate than those of whole genome shotgun (WGS) assemblies, which are generated de novo with no supporting information.<br><br>To address this concern, we have refocussed the Introduction and Discussion to make clear that the Fosill 40kb mate pair mapping approach described in the paper assesses shorter sequence assembly scales and not longer, pseudo-molecule scale, assemblies. The title has also been edited to add the word "sequence" before assemblies in order show that 40 kb long mate-pairs are used to assess the fidelity of sequence contigs and scaffolds on the scale of 100-1000 Kb. This is the contig and scaffold size range of sequence assembly scales from both WGS and BAC-based sequence assemblies- from 100-1000kb. So, we think it is reasonable to compare the different assemblies at this scale. Similar approaches have been taken in assessing different human genome sequence assemblies. It's important to note however that assembly errors at these shorter scales can be propagated into larger-scale errors unless they are detected by eg HiC, linked reads, or optical mapping. Hence it is timely, at this stage of wheat genomics, to assess sequence assembly scale differences using a common approach.<br><br>Our primary motivation for assessing the fidelities of sequence assemblies is to help guide the next steps of sequencing technology for wheat, hence the focus on sequence assembly and scaffold accuracy. We did not set out to assess larger-scale fidelity as 40 kb mate pairs are just not suitable for this. We hope the revised manuscript makes our objectives clearer. For example, in the Discussion we suggest that HiC, linked reads and perhaps Nanopore long reads can be used to generate and assess longer scale assemblies.<br><br>We believe we have addressed the issues raised and, as Reviewer x comments, the findings described in the paper would "be extremely useful" for guiding assembly strategies.<br><br>Reviewer 1<br><br>This reviewer states that the work described is a timely contribution to wheat genome assembly work and states that the paper would be suitable for publication subject to specific edits. These have been done as follows:

Powered by Editorial Manager® and ProduXion Manager® from Aries Systems Corporation

	1. corrected to "avoid"<br>2. (parameter)- deleted<br>3. corrected to "were mapped"<br>4. sd = standard deviation defined<br>5. corrected to "publicly"<br>6. Num> n50 now spelt out in Table 3B<br><br><br>Reviewer 3<br><br>This reviewer finds the work to be interesting and provides readers with useful information for guiding genome assembly.<br><br>Specific comments<br><br>A detailed description of how to construct a Fosill library is provided in Additional File 2. This describes steps from DNA preparation through to specific modifications for wheat.<br><br>Regarding SMRT reads, the manuscript has been edited again to make sure that SMRT reads formed part of the independent Triticum v 3.0 assembly generated by Alexi Zimin and colleagues. We did not use SMRT reads to assess assemblies. Considering whether Fosill can "exist together" or be used together with Nanopore /PacBio sequences to create improved assemblies, the work described in this manuscript indicates that such long reads need to be very accurate to avoid mis-assembly. In the case of Nanopore sequences, these have, to the best of our knowledge, not yet been used in wheat assemblies. SMRT sequences as used in the Triticum 3.0 assembly must be fully corrected with Illumina sequences before they can be used to create accurate assemblies. Once this is done then Fosill mate-pair reads can be incorporated with accurate long reads in wheat assemblies. In fact, Fossil libraries are very hard to make at sufficient coverage, so it's likely that they will not be generally used in future wheat assemblies.<br><br>L90-95<br>Here in the Background we aim to provide a brief summary of how two large and challenging genomes of pine and wheat have been sequenced. Long reads have been very important for generating good assemblies, therefore their use does need to be described. Hence our desire to assess the fidelity of these different assemblies. The Background section has been re-organised and edited to help clarity.<br><br>L138<br>Physical and sequence coverage calculations are now shown in Additional File 2.<br><br>Table 1 has been edited so that all values are to the first decimal point<br><br>Additional File 1, Table 3 and Figure 5.<br><br>Here 0 x coverage means that the simulation used no 40kb mate-pairs in the simulation. So it does mean "no data". The legend to Table 3 in Additional File 1 has been modified to explain what 0 means.
**Additional Information:**	
**Question**	**Response**
Are you submitting this manuscript to a special series or article collection?	No
**Experimental design and statistics**	Yes

Powered by Editorial Manager® and ProduXion Manager® from Aries Systems Corporation

Full details of the experimental design and statistical methods used should be given in the Methods section, as detailed in our Minimum Standards Reporting Checklist. Information essential to interpreting the data presented should be made available in the figure legends.<br><br>Have you included all the information requested in your manuscript?	
**Resources**<br><br>A description of all resources used, including antibodies, cell lines, animals and software tools, with enough information to allow them to be uniquely identified, should be included in the Methods section. Authors are strongly encouraged to cite Research Resource Identifiers (RRIDs) for antibodies, model organisms and tools, where possible.<br><br>Have you included the information requested as detailed in our Minimum Standards Reporting Checklist?	Yes
**Availability of data and materials**<br><br>All datasets and code on which the conclusions of the paper rely must be either included in your submission or deposited in publicly available repositories (where available and ethically appropriate), referencing such data using a unique identifier in the references and in the "Availability of Data and Materials" section of your manuscript.<br><br>Have you have met the above requirement as detailed in our Minimum Standards Reporting Checklist?	Yes

Powered by Editorial Manager® and ProduXion Manager® from Aries Systems Corporation

Manuscript

Click here to download Manuscript Fosill jumping libraries_V8.docx

Independent assessment and improvement of wheat genome sequence assemblies using Fosill jumping libraries.

Fu-Hao Lu[1]*, Neil McKenzie[1]*, George Kettleborough[2], Darren Heavens[2], Matthew D Clark[2], Michael W Bevan[+1]

[1]John Innes Centre, Norwich Research Park, Norwich NR4 7UH, UK

[2]The Earlham Institute, Norwich Research Park, Norwich NR4 7UZ, UK

*Joint first Authors

[+]Corresponding Author

ORCID details: Fu-Hao Lu: 0000-0003-3783-4685. Neil McKenzie: 0000-0001-9818-9216. George Kettleborough: 0000-0003-2606-0063. Darren Heavens: 0000-0001-5418-7868. Mathew D. Clark: 0000-0002-8049-5423. Michael W Bevan: 0000-0001-8264-2354.

Abstract

Background

The accurate sequencing and assembly of very large, often polyploid, genomes remain a challenging task, limiting long-range sequence information and phased sequence variation for applications such as plant breeding. The 15 Gb hexaploid bread wheat genome has been particularly challenging to sequence, and several different approaches have recently generated long-range assemblies. Mapping and understanding the types of assembly errors is important for optimising future sequencing and assembly approaches and for comparative genomics.

Results

Here we use a Fosill 38 Kb jumping library to assess medium and longer–range order of different publicly available wheat genome assemblies. Modifications to the Fosill protocol generated longer Illumina sequences and enabled comprehensive genome coverage. Analyses of two independent BAC-based chromosome-scale assemblies, two independent Illumina whole genome shotgun assemblies, and a hybrid Single Molecule Real Time (SMRT-PacBio) and short read (Illumina) assembly were carried out. We revealed a surprising scale and variety of discrepancies using Fosill mate-pair mapping and validated several of each class. In addition, Fosill mate-pairs were used to scaffold a whole genome Illumina assembly, leading to a three-fold increase in N50 values.

Conclusions

Our analyses, using an independent means to validate different wheat genome assemblies, show that whole genome shotgun assemblies based solely on Illumina sequences are significantly more accurate by all measures compared to BAC-based chromosome-scale assemblies and hybrid SMRT-Illumina approaches. Although current whole genome assemblies are reasonably accurate and useful, additional improvements will be needed to

generate complete assemblies of wheat genomes using open-source, computationally efficient and cost-effective methods.

Keywords

Wheat genome; genomics; assembly methods; Fosills; long-range genome assembly; Illumina; PacBio

Background

Genome sequence assemblies are key foundations for many biological studies, therefore the accuracy of sequence assemblies is a fundamental prerequisite for their use. Multiple types of differences in the information content of DNA molecules, from single nucleotide polymorphisms (SNPs) to large-scale structural variation (SV), form part of natural genetic variation that can cause phenotypic variation [1,2]. Distinguishing such *bona fide* variation from apparent variation generated by sequence and assembly methods is therefore a critically important activity in genomics.

Sequence assemblies are generally incomplete and contain multiple types of errors, reducing their information content. Gaps in assemblies can occur where no sequence reads were generated for that region, but this is now increasingly unlikely given the very deep coverage achievable by short read sequencing, improved sequence chemistry, and template preparation methods that avoid bias, such as that introduced by PCR [3]. Closely related repetitive DNA sequences can lead to incorrect joins in assemblies, or to an unresolvable assembly graph that breaks an assembly. Assemblies can be either joined or broken inadvertently by closely related or polymorphic sequences that cause alternate, multiple, or collapsed assemblies, for example in assemblies of polyploid genomes [4]. Errors and incompleteness can also obscure important genomic information such as the correct order (phasing) of sequence variants.

A broad spectrum of sequence and assembly artefacts can be distinguished from natural sequence variation, structural variants identified, and sequence variation phased, using long-range sequence information. Sequences of long single molecules generated by PacBio Single Molecule Real Time (SMRT) and Nanopore technologies are increasingly used for *de novo* genome assembly [5,6]. Linked read technologies such as 10X Genomics reads also have great promise for long-range ordering of scaffolds assembled from short reads, for defining extended haplotypes, and for identifying structural variation [7]. Chromatin interaction frequency data is also used to position sequence scaffolds into larger chromosome-scale pseudomolecular assemblies [8], while optical and genetic maps provide complementary ways of assessing chromosome- scale pseudomolecule assemblies [9]. Nevertheless, assessing sequence assemblies across smaller scales (100-1000 Kb) remains an essential task, as such local assembly errors can be propagated into larger pseudomolecule-scale alignments.

These improvements in sequencing and assembly are enabling the creation of genomic resources for very large and complex genomes, including those of grasses and gymnosperm trees, which have massive repetitive DNA tracts comprising about 80% of their genomes. The 22 Gb genome of loblolly pine (*Pinus taeda*), initially assembled from Illumina paired end sequence reads [10], has been significantly improved using SMRT sequencing [11]. 10X Genomics linked reads were used to generate an eight-fold increase in scaffold NG50 sizes of sugar pine (*P. lambertiana*) genome assemblies to nearly 2 Mb [12]. Bread wheat (*Triticum aestivum*) has a large 15 Gb allohexaploid genome comprising three closely-related and separately maintained A, B and D genomes [13]. BAC-based physical maps of flow-sorted chromosomes have been made to avoid mis-assembly of the separate genomes [14]. A 790 Mb tiling path of BACs from chromosome 3B has been sequenced and assembled [15], and represents the most complete available chromosome-scale assembly of wheat to date. Whole genome shotgun (WGS)[16] purified chromosome sequencing [17]

approaches to wheat genome assembly have also been taken, but these assemblies were very fragmentary and incomplete. Recently a new approach to template preparation and assembly generated separate assemblies of the A, B and D genomes, but although more complete, these remain quite fragmentary [18]. A near-complete and highly contiguous assembly of Illumina paired-end and mate-pair reads from wild emmer wheat (WEW, *Triticum turgidum ssp dicoccoides*), a tetraploid progenitor of bread wheat, has also recently been published [19], suggesting this approach also has promise for sequencing hexaploid wheat genomes. Finally, long SMRT sequence reads integrated with Illumina sequence coverage increased the size and contiguity of maize [20], a diploid wheat progenitor [21] and hexaploid wheat genome assemblies [22]. It is therefore timely to assess the extent of different assembly error types in each of these different tetraploid and hexaploid wheat assemblies.

Generating accurate genome assemblies is essential for identifying haplotypes selected by breeders and for mapping large-scale structural variation contributing to agronomic performance [23]. Therefore, assessing the fidelity of wheat sequence assemblies generated by different strategies using a common approach is important for both determining optimal sequencing and assembly strategies and for identifying structural variation. Here we use very long 40 Kb mate-paired sequences of wheat fosmid clones to assess three different wheat whole genome assemblies and two BAC-based wheat chromosome assemblies. Our analyses have identified a range of error types in all assemblies and identify more optimal approaches to wheat genome assembly. We also integrated fosmid end-sequences into whole genome assembly scaffolds, and substantially increased scaffold sizes of both fragmentary and more contiguous assemblies.

Results

Creating and assessing a wheat fosmid clone library

Fosmid clone libraries have been used to assess genome assemblies and identify structural variation in human [24,25] and pine genomes [11]. Fosmids are used because DNA is cloned in a precise range of 38±3 Kb by efficient packaging in phage lambda and cohesive end circularisation. Fosmid clone inserts have been converted to Illumina sequencing templates to generate 38 Kb mate-pair “jumping libraries” and used to improve assemblies of the mouse genome [26]. In genomes with extensive tracts of very similar repeats, and with closely-related homoeologous chromosomes that have been challenging to assemble, fosmid jumping libraries could provide an independent means to assess the fidelity of different wheat genome assemblies and to improve them. In particular, precisely-spaced 38 Kb paired sequences can identify a range of local assembly errors over scales that include current contig and scaffold sizes of wheat BAC and WGS assemblies.

To explore the potential of fosmid jumping libraries for assessing and improving different tetraploid and hexaploid wheat sequence assemblies, we first carried out a simulation of whole genome shotgun assembly of three long 3.5 - 4.1 Mb scaffolds of wheat chromosome 3B generated by sequencing and assembling a manually curated physical map of BACs [15]. Simulation settings used different paired-end distances, read lengths and sequence coverage on faux reads from chromosome 3B to assess how read-depth and read-length of 38 Kb mate-paired reads, contributed to re-assembly of wheat scaffolds (Additional File 1). Addition of 38 Kb mate-pair reads was required for accurate and complete reconstruction of all three scaffolds under simulation conditions. Paired-end read lengths between 100 – 250 bp were then assessed using a common combination of mate pair distances and sequence coverage. Reads of over 200 bp were required for consistent re-assembly of all three scaffolds. Finally, simulation of sequence coverage of 38 kb mate pair reads of length 250 bp showed that consistent re-assembly of all three scaffolds required sequence coverage of at least 0.75x (Additional File 1). Taken together, these simulations showed that 38 Kb paired-end 250 bp reads with a sequence coverage of approximately 0.75x (>50x physical coverage, Additional File 2) could be used to guide and assess assemblies of the wheat genome.

The FosiII vector system was developed for converting fosmid clones to Illumina paired-end read templates [28]. We modified this FosiII conversion protocol to generate long paired-end 250 bp Illumina reads, to maximise library complexity, and to minimise clonal- and PCR-based amplification bias. Both of these modifications were required to maximise unique matches of paired-end reads to the highly repetitive polyploid wheat genome, and to maximise sequence coverage of the large genome. Additional File 2 describes modified protocols for library preparation and paired-end read analyses. These involved increasing the time of nick-translation to between 50-60 minutes on ice to generate inverse PCR products with a peak size distribution of 785-860 bp (Additional File 2). This minimised overlap of 250bp reads from either end of the PCR product. For each pool of 5-10M FosiII clones, a small sample of the circularised template was amplified for up to 16 cycles, and the minimum number of cycles required (generally 12-13) to generate sufficient template for sequencing was estimated.

Table 1 in Additional File 2 summarises the FosiII libraries produced and the paired-end sequences generated from them. Paired-end reads that overlapped each other on the template were discarded (2.61%), while 11.91% of the raw reads were excluded after vector/adapter sequence and quality trimming. The final number of 576 M paired-end sequences (85.5% of the total reads) were generated from 54.61M FosiII clones (1.8 sequence coverage, 138x physical coverage). These were then mapped to the chromosome 3B pseudomolecule to measure the insert size distribution of the libraries (Additional File 3). Figure 1A shows the size distribution of 588,268 mapped read pairs, which had a mean estimated insert size of approximately 37,725 bp. This is the expected insert size range in the FosiII4 vector [28], and demonstrated successful size selection during packaging. Figure 1B shows the distribution of mate-pairs mapped in 100 kb windows across chromosome 3B BAC pseudomolecule. Reads with a depth of ″5 covered 494 Mb of the total 833 Mb

chromosome, accounting for 59% of the chromosome sequence. Their even distribution across the pseudomolecule indicated that the libraries were representative of the entire chromosome. There were approximately 30 distinct peaks of greatly increased read-depth (Figure 1B) in the 100 kb windows across chromosome 3B. These probably correspond to mate-pairs spanning approximately 40 Kb repeated regions common to multiple genomic loci. These reads accounts for 80% of the alignments but covered only 4.3% of chromosome 3B. For all subsequent analyses only Fosill mate-pairs of sequence depth ″5 were used. Finally, reads that mapped to multiple locations, which lacked a paired read in the expected genomic location, or which had a paired read in the incorrect orientation, were removed.

Using Fosill mate pairs to assess wheat chromosome sequence assemblies

The even representation of long mate-paired reads across the chromosome 3B pseudomolecule indicated their suitability for assessing wheat sequence assemblies and for making new joins in wheat sequence scaffolds. For assessing assemblies, a windows-based filter was developed to identify sets of ≥5 unique neighbouring Fosill sequence reads in a “driver” window of <10 kb and their ≥5 mate-pair reads in a “follower” window of <20 kb on chromosome and genome assemblies. The vast proportion of mate-paired reads fell within this distance distribution (Additional File 3, Figure 1). Using this approach to map Fosill reads, we aimed to identify different types of paired-end matches to genome sequence assemblies. These can be used to identify genome assemblies consistent with the 37.7 Kb mate-pair distances +/- sd, to identify possible new joins between assemblies, and to identify different types of inconsistencies in the range of current publicly available wheat genome

assemblies. Figure 2A illustrates the possible types of Fosill paired-end matches to assemblies.

Tables 1A-1E show the outcomes of mapping Fosill paired-end reads to BAC-based bread wheat chromosome assemblies of chromosome 3B [15], TGACv1 Illumina assemblies of 3B [18], the Triticum 3.0 whole genome assembly of Pacbio SMRT and Illumina sequences of chromosomes 3B and 3DL (Additional Files 4 and 5) [22], and DeNovoMagic assemblies of Illumina sequences from wild emmer wheat (WEW) chromosome 3B [19]. We also assessed an assembly of hexaploid wheat chromosome 3DL from sequenced BACs in a minimal tiling path using an automated pipeline (Additional File 6). Finally, a set of larger whole genome assemblies of the TGACv1 Illumina wheat genome were also assessed. These assemblies represent diverse approaches to sequencing wheat chromosomes and chromosome arms, including manually curated and automated BAC-based assemblies, two different Illumina-based assembly methods, and a combined Illumina and Pacific Biosciences SMRT assembly of wheat chromosomes.

Variation in Fosill insert sizes were consistent across the TGACv1, Triticum 3.0 and DeNovo Magic whole genome assemblies of chromosome 3B and the 3DL BAC assemblies. In contrast, chromosome 3B BAC assemblies had a higher variation of insert sizes (Table 1A). This may be due to a higher proportion of mis-assemblies in the 3B BAC assembly that could have introduced or removed small tracts of sequences, and possibly due to the use of a mixture of 454 and Illumina sequences in the assembly. This variation in Fosill mate-pair matches did not contribute to assessment of assembly accuracy. The accuracy of assemblies was estimated by counting the bases included in correctly-sized windows (mean insert size +/-sd) of Fosill mate-pair reads, and by the proportion of assemblies/scaffolds that were fully consistent with Fosill mate-pair windows along their length. The un-edited BAC-based scaffolds of chromosome 3DL were the least accurate, with only 17% of the assemblies covered with consistent fossil mate-pair matches, and 57.2% of the sequence

included under consistent mate-pair matches (Table 1A). The 3B BAC assemblies, which were extensively manually edited, were considerably more accurate, with 66% consistent assemblies and 85.7% of sequences in consistent windows. Looking at the TGACv1 3B assemblies, 61% of scaffolds were consistent and 80.5% of sequences were contained within consistent Fosill windows. In contrast, larger TGACv1 assemblies from the whole genome were all consistent with mate-pair windows and 98.9% of the sequences were in consistent windows. These differences between TGACv1 3B assemblies are most likely due to the inclusion of many shorter assemblies in the 3B assembly that limit the potential for 38 Kb mate-pair mapping, for example, there will be a low proportion of matches at the ends of assemblies. The Triticum 3.0 WGS assembly of 3B had 92% consistent assemblies, and 91% of sequences within consistent Fosill windows. Similarly, the Triticum 3.0 WGS assembly of chromosome 3DL had 99.5% assemblies and 86.2% of sequences in consistent windows. The DeNovo Magic WGS assembly of *T. turgidum* 3B contained 99.6% of sequences in consistent Fosill windows. As these assemblies were integrated into a single pseudomolecule the measure of the number of correct scaffolds was 100%.

Four different classes of discrepancies that may be due to assembly problems were assessed using Fosill mate pair mapping to assemblies: failed scaffolding, in which scaffolds had matches to only one end of Fosill end-sequences, and which may need to be broken; orientation errors in which the direction of one region of a scaffold is consistently reversed with respect to flanking regions; insertions, in which the span of Fosill mate-pair matches is greater than expected; and deletions, in which mate-pair spans are less than expected. These results are summarised in Tables 1B-1E. Of these potential error types, the most frequent were the potential erroneous joining of assemblies. These were highest in the BAC assemblies of hexaploid wheat, and lowest in the DenovoMAGIC assembly of WEW 3B. An example of this is shown in Figure 1B, where two BAC-based scaffolds were assembled at either end of chromosome 3B. Fosill mapping evidence, supported by TGACv1 assemblies, showed that the two scaffolds can be merged in opposite orientation to that originally

assembled. Figure 2C reveals a 12 Kb deletion in a TGACv1 assembly that was due to a missing tandem duplication of the repeat, as validated by comparison with the Triticum 3.0 assembly. An aberrant insertion in a TGACv1 scaffold identified by Fosill mate-pair mapping was also validated by comparison with the Triticum 3.0 assembly (Figure 2D).

The TGACv1 large assemblies have relatively low numbers of mis-assemblies. The Triticum 3.0 assemblies of both 3B and 3DL had a consistently large number of potential mis-assemblies, with about 400-500 per chromosome or chromosome arm, affecting about 10 Mb of sequence region. Potential deletion errors, in which assemblies may be missing sequences, were most frequent in the BAC assembly of chromosome 3B, and were also the most frequent type of error in the DenovoMAGIC assembly. Deletions were least frequent in the TGACv1 whole genome assembly. Potential erroneous insertions were less frequent than deletions, with the highest rates of both types of potential error in BAC-based assemblies. In general, potentially erroneous deletions were more common in all assemblies than insertions. Mis-orientations were the rarest potential error type, were most prevalent in manual assembled 3B BAC scaffolds, were essentially absent from TGACv1 and Triticum 3.0 assemblies, but were more frequent in the DeNovo Magic WEW 3B assembly.

Using Fosill mate-pairs to create more contiguous assemblies

The wheat Fosill library was also used to create new joins in different assemblies. Table 2A shows that Fosill mate-pair reads made 267 new links between 477 chromosome 3B BAC scaffolds. Where available, TGACv1 3B assemblies spanning the new links precisely (124 cases), supporting the new join, and no examples were found where the new Fosill joins linked the wrong neighbours or the wrong strand. We then applied the Fosill mate pairs to make new joins in chromosome 3B TGACv1 assemblies and chromosome 3DL BAC assemblies. Table 2B shows the total assembly sizes were increased, while the number of scaffolds in the assemblies was decreased, and the scaffold n10 more than doubled in size. This showed, as predicted by simulations (Figure1), that 38 Kb mate-pair reads can make

new links that substantially improve contiguity of both WGS and BAC-based assemblies. Where available, independent assemblies supported these new Fosill-based links. Figure 3 shows the distribution of scaffold sizes and numbers before and after Fosill linking on TGACv1 chromosome 3B (panel A) and chromosome 3DL BAC (Panel B) assemblies. Increases in the numbers of larger assemblies and concomitant reduction in the numbers of smaller assemblies after Fosill joining was more apparent in the chromosome 3B WGS scaffolds than in the 3DL BAC scaffolds. This may reflect the fewer joins needed in the less fragmentary 3B assembly (2,808 scaffolds) that the very fragmented 3DL assembly (23,433 scaffolds).

Based on these improvements in both BAC- based and WGS scaffold contiguity by integrating Fosill mate-pair reads, we re-scaffolded the complete TGACv1 WGS assembly of the wheat variety Chinese Spring 42 [19]. Figure 4 and Additional File 7 show the scaffold sizes of each chromosome arm before and after integration of Fosill mate-pairs in the new TGACv2 assembly. Substantial increases in scaffold N50 of between 2.7- 3.2-fold were achieved. The largest scaffolds increased is size between 1.5- 3.2-fold, with the largest scaffold of 2.8 Mb on chromosome 3B.

Discussion

Bread wheat is one of the three major cereals that we depend on for our nutrition, and generating accurate long-range assemblies is essential for new genomics-led approaches to crop improvement. However, its genome has been exceptionally challenging to sequence due to its polyploid composition of three closely-related large genomes, and extensive tracts of very similar repetitive sequences. Two strategies have been followed to deal with this genomic complexity: the first used BAC clones made from purified chromosomal DNA to reduce the complexity of chromosome-specific assemblies [15]; the second set of approaches uses different types of whole genome shotgun sequence technologies and

assembly methods [18,19,22]. At this stage of wheat genome sequencing, when assemblies from these complementary and contending approaches are available for use, it is timely to assess the accuracy of these different assemblies. For this, we mapped precise 38 Kb Fosill long mate pair reads to measure errors in different assemblies of chromosome 3B and the long arm of chromosome 3DL. We also used these Fosill mate pair reads to increase whole genome assembly contiguity.

In order to maximise the accuracy of Fosill mate-pair read mapping to the A, B or D genomes and to repetitive regions of the hexaploid wheat genome, we modified the template conversion protocol of the Fosill 4 vector system [26] to generate longer paired 250 bp Illumina sequence reads. Nick-translation reactions to extend Nb.BbvCI nicks were optimised to generate an Illumina sequencing template between 750 - 1,000 bp. PCR amplification of re-circularised products was optimised to reduce amplification to the minimum required for efficient sequencing of a large library. Overall, 576.5M read pairs were generated from 55.1M clones (Additional File 2), generating 1.8x total sequence coverage of the wheat genome. When reads were mapped to chromosome 3B sequence assemblies a consistent size distribution around 37.7 Kb was observed (Figure 1A), demonstrating correct phage lambda packaging and processing. Read depth varied several thousand-fold along chromosome 3B, likely due to matches of read-pairs to highly repetitive regions from across the genome. Consequently, only read-pairs with depth ≤5 were used. Using this filter, we obtained sequence coverage of nearly 60% of the 833 Mb BAC-based chromosome 3B assembly. Simulations indicated that 0.75x sequence coverage of paired-end 250 bp reads was effective in creating long-range assemblies of wheat (Additional File 1), therefore we used Fosill read mapping for subsequent analyses.

Fosmid mate-pair sequences have been used to close gaps [27] and detect structural variation [24] in the human genome. Different types of variation, including insertions, deletions and inversions spanning tens to hundreds of Kb were detected. Larger scale errors, for example generated in the creation of pseudomolecules spanning chromosomes, are more readily detected using optical mapping and chromatin conformation methods [28]. Fosill reads were mapped to different assemblies of chromosome 3B and the long arm of chromosome 3D in order to compare the full range of current publicly available hexaploid and tetraploid wheat assemblies. Tables 1A-1E) show the types of inconsistencies detected in different wheat assemblies using this approach. Looking first at the proportion of bases in different assemblies that were fully consistent with mapped 38 Kb mate-pair reads (Table 1A), the DenovoMAGIC Illumina-based WEW assembly, the larger TGACv1 assemblies, and the merged SMRT long-read and Illumina short read Triticum 3.0 assembly of chromosome 3B had respectively 99.6%, 98.9% and 91.0% of bases in consistent Fosill windows. The manually curated BAC-based assembly of 3B had 85.7% of consistent bases, while the TGACv1 3B assembly had 80.5% of assembled sequence in consistent windows. The difference between the total TGACv1 3B assembly probably reflects the inclusion of shorter assemblies in the TGACv1 3B assembly that are shorter than 38 Kb. The non-curated BAC assembly of chromosome 3DL was the least accurate according to this measure, with only 57.2% of bases in consistent windows. These data demonstrate the superior accuracy of *de novo* whole genome sequencing strategies that incorporate deep and long 250 bp Illumina paired-end and mate-pair sequence coverage, and the relative accuracy of long-range assemblies generated by mate-pair assembly strategies [18,19], compared to BAC-based and SMRT strategies [15,22].

The most frequent type of inconsistency identified by Fosill mapping was the potential incorrect joining of assemblies (Table 1B). Illumina strategies using long mate-pair information produced the fewest incorrect joins, while BAC-based assemblies produced the

most. Interestingly, the hybrid Triticum 3.0 assembly of both 3B and 3DL made from PacBio SMRT reads combined with 150 bp Illumina paired end reads [11] had more potential assembly errors than the Illumina- only assemblies, with 517 and 444 potential mis-assemblies on chromosomes 3B and 3DL respectively. While the merged Triticum 3.0 assembly is more complete and contains no unknown bases, it was based on relatively short Illumina reads (150 bp paired end reads vs 250 bp paired end reads in the TGAC v1 and DenovoMAGIC assemblies) and did not include any longer Illumina mate-pair sequences. The multiple merging steps used in generating the Triticum 3.0 assembly may also contribute to the relatively high numbers of mis-assemblies. Assembly methods may also need further optimisation to utilize fully the potential of SMRT long reads. Furthermore, integrating long 250 bp Illumina reads into mega-reads may improve assemblies by distinguishing very closely related sequences, such as repeat regions from homoeologous chromosomes.

Potential deletion events were also quite common in all assemblies and were the most common inconsistencies detected in DenovoMAGIC assemblies of 3B. The sizes of these events are not known precisely, but they have a minimum size of 12 Kb (Table 1E). These probably arise from missing tracts of near-identical sequence in assemblies. Similarly, potential insertions may arise from the incorrect integration of near-identical sequences into assemblies. The observation that potential deletions are more frequent than potential insertions suggests that all WGS-alone assembly strategies could achieve more complete assemblies of the wheat genome, such as that achieved using PacBio SMRT sequence assemblies. Finally, potential mis-orientations/inversions of assemblies are more common in the DevoMAGIC assembly of 3B that the other whole- genome assemblies. Although this approach has yet to be fully described, mis-orientations may reflect more relaxed criteria for linking scaffolds than related Illumina-based assembly and scaffolding approaches [18].

How much more accurate can the best current assemblies of bread wheat and wild emmer wheat be, judging by their assemblies of chromosome 3B? Fosmid end-mapping to 2005 versions of human genome assemblies [24] identified 297 longer range discrepancies in the 3.2 Gb genome. Scaling from chromosome 3B (0.8 Gb) with 127 potential inconsistencies, our analyses predict 480 discrepancies per 3Gb of wild emmer wheat genome assembly, roughly twice the error frequency of 2005 versions of the human genome. It is likely that a DenovoMAGIC version of the hexaploid bread wheat genome will achieve similar high levels of accuracy and coverage.

Three-fold increases in the scaffold N50 sizes of the TGACv1 whole genome assembly were achieved by an additional scaffolding step using Fosill mate-pairs. In addition to making a more useful genomic resource, this additional scaffolding shows the relatively fragmentary but highly accurate TGACv1 assembly has the potential for substantial further improvement as an open-source and computationally efficient approach to assembling multiple wheat genomes [18]. For example, the direct integration of linked read technologies [29] and Nanopore long reads [30] into this assembly process should substantially increase contiguity, and directly and precisely identify a wide range of structural and phased sequence variation in wheat genome assemblies that are required for trait analyses and accelerating breeding.

Methods

Detailed descriptions of experimental and computational procedures are shown in Additional Files. These describe simulation of 38 Kb mate-pair reads for assembly (Additional File 1), Production and sequencing of Fosill libraries (Additional File 2) and physical mapping and sequencing of BACs from chromosome 3DL (Additional File 3).

General bioinformatics

All analytical pipelines have been deposited in GitHub and in SciCrunch (NGSimple, RRID:SCR_016165; ReadCleaner4Scaffolding, RRID:SCR_016166). Relevant links are shown in the manuscript and Additional Files. Joinable read pairs from Illumina Miseq or HiSeq sequencing were removed using FLASH v1.2.11 [31]. Ligation adaptors in reads were trimmed off using CutAdapt v1.6 [32]. Sequencing primer sequences and low-quality sequences in reads were removed using Trimmomatic v0.32 [33]. The resulting reads were evaluated using FastQC v1.2.11 [34].

Trimmed reads were further filtered using ReadCleaner4Scaffolding pipeline (https://github.com/lufuhao/ReadCleaner4Scaffolding). Both mates in each pair were mapped to chr3B BAC scaffolds using bowtie v1.0.1 (Bowtie, RRID:SCR_005476)[35]. And then the Picard MarkDuplicates v1.108 (Picard, RRID:SCR_006525)[36] was used to remove the duplicates as single reads. A read depth threshold was used to remove the repeat-like reads by plotting the summary of output from samtools depth, and the reads mapped to those regions with higher depth were not used for scaffolding. The remaining reads were subjected to removal again as pairs.

Those reads mapped to multiple positions, whose mates were not mapped, or had the wrong orientation, were removed. A window size filter was applied to identify sets of ≥5 neighbouring reads in sliding windows of less than 10 Kb that had all their mates in a following window of less than 20 kb. Variations of the expected distance between mate-pairs (average ± standard deviation, sd) of approximately 3 sd was used to identify potential assembly discrepancies.

Abbreviations

BAC: Bacterial Artificial Chromosome; BBSRC: Biological and Biotechnological Sciences Research Council; bp: base pair; Gb: Giga basepair; Kb: Kilo basepair; Mp: Mega basepair; sd: standard deviation; SMRT: Single Molecule Real Time; SNP: single nucleotide

polymorphism; SV: structural variation; WEW: wild emmer wheat; WGS: whole genome shotgun.

Availability of supporting source code and requirements

Project names: NGSimple, & ReadCleaner4Scaffolding

Project home pages: https://github.com/lufuhao/NGSimple & https://github.com/lufuhao/ReadCleaner4Scaffolding

Operating system: Linux

Programming language: Perl, Bash

Other requirements: See bash scripts in ProgramRoot/utils/

License: GNU GPL v3

RRID: NGSimple, RRID:SCR_016165; ReadCleaner4Scaffolding, RRID:SCR_016166

Data Availability

Fosill mate-pair sequence reads from *Triticum aestivum* Chinese Spring 42 generated in this study have been submitted to the EBI European Nucleotide Archive (ENA) and are available in study accession PRJEB23322. The TGAC v2 *Triticum aestivum* Chinese Spring 42 whole genome assembly is available in ENA study accession PRJEB23893. *Triticum aestivum* Chinese Spring 42 chromosome 3DL BAC scaffold sequences are available in ENA study accession PRJEB23358. Snapshots of the pipeline and data are available in the *GigaScience* GigaDB repository[37].

Additional Files

Additional File 1. Genome Assembly Simulation.doc

Additional File 2. Fosill Library Production.doc

Additional File 3. Fosill Mate-Pair Mapping.doc

Additional File 4. Triticum 3.1 PacBio assemblies of chromosome 3B.xls

Additional File 5. Triticum 3.1 PacBio assemblies of chromosome 3DL.xls

Additional File 6. Sequencing Chromosome 3DL BAC Minimal Tiling Path.doc

Additional File 7. TGACv2 chromosome scaffolds.xls

Declarations

The authors declare they have no competing interests.

Funding

This work was supported by a Biological and Biotechnological Sciences Research Council (BBSRC) strategic LOLA award to MWB (BB/J00328X/1 and MDC (BB/J003743/1), The FP7 Triticeae Genome Project to MWB, and a BBSRC Institute Strategic Programme Grant (GEN) BB/P013511/1 to MWB. BBSRC Institute Strategic Programme Grant (BB/J004669/1) and Core Strategic Programme Grant (BB/CSP17270/1) also supported work at the Earlham Institute. Sequencing was delivered via the BBSRC National Capability in Genomics (BB/J010375/1) at the Earlham Institute and performed by members of the Genomics Pipelines Group.

Authors' contributions.

MWB conceived and coordinated the project, and wrote the manuscript. F-HL planned and carried out bioinformatics analyses, NMcK constructed the Fosill libraries, GK and MDC sequenced chromosome 3DL BACs and managed sequence data, and DH managed all sequencing library production and Illumina sequencing.

Acknowledgements

We are grateful to Louise Williams (Broad Institute) for Fosill vectors and detailed advice.

References

1. Botstein D, Risch N. Discovering genotypes underlying human phenotypes: past successes for mendelian disease, future approaches for complex disease. Nat Genet. 2003;33 Suppl:228–37.

2. Schneeberger K, Ossowski S, Ott F, Klein JD, Wang X, Lanz C, et al. Reference-guided assembly of four diverse Arabidopsis thaliana genomes. Proc Natl Acad Sci U S A. 2011;108:10249–54.

3. Weisenfeld NI, Yin S, Sharpe T, Lau B, Hegarty R, Holmes L, et al. Comprehensive variation discovery in single human genomes. Nat Genet. 2014;46:1350–5.

4. Chaisson MJP, Wilson RK, Eichler EE. Genetic variation and the de novo assembly of human genomes. Nat Rev Genet. 2015;16:627–40.

5. Chin C-S, Peluso P, Sedlazeck FJ, Nattestad M, Concepcion GT, Clum A, et al. Phased diploid genome assembly with single-molecule real-time sequencing. Nat Methods. 2016;13:1050–4.

6. Jain M, Koren S, Quick J, Rand AC, Sasani TA, Tyson JR, et al. Nanopore sequencing and assembly of a human genome with ultra-long reads. Nat Biotechnol. 2018 Apr;36(4):338-345. doi: 10.1038/nbt.4060.

7. Zheng GXY, Lau BT, Schnall-Levin M, Jarosz M, Bell JM, Hindson CM, et al. Haplotyping germline and cancer genomes with high-throughput linked-read sequencing. Nat Biotechnol. 2016;34:303–11.

8. Kaplan N, Dekker J. High-throughput genome scaffolding from in vivo DNA interaction frequency. Nat Biotechnol. 2013;31:1143–7.

9. Beier S, Himmelbach A, Colmsee C, Zhang X-Q, Barrero RA, Zhang Q, et al. Construction of a map-based reference genome sequence for barley, Hordeum vulgare L. Sci Data. 2017;4:170044.

10. Neale DB, Wegrzyn JL, Stevens KA, Zimin AV, Puiu D, Crepeau MW, et al. Decoding the massive genome of loblolly pine using haploid DNA and novel assembly strategies. Genome Biol. 2014;15:R59.

11. Zimin AV, Stevens KA, Crepeau MW, Puiu D, Wegrzyn JL, Yorke JA, et al. An improved assembly of the loblolly pine mega-genome using long-read single-molecule sequencing. Gigascience. 2017;6:1–4.

12. Crepeau MW, Langley CH, Stevens KA. From Pine Cones to Read Clouds: Rescaffolding the Megagenome of Sugar Pine (Pinus lambertiana). G3 . 2017;7:1563–8.

13. Marcussen T, Sandve SR, Heier L, Spannagl M, Pfeifer M, The International Wheat Genome Sequencing Consortium, et al. Ancient hybridizations among the ancestral genomes of bread wheat. Science. 2014;345:1250092.

14. Šafář J, Bartoš J, Janda J, Bellec A, Kubaláková M, Valárik M, et al. Dissecting large and complex genomes: flow sorting and BAC cloning of individual chromosomes from bread wheat. Plant J. 2004;39:960–8.

15. Choulet F, Alberti A, Theil S, Glover N, Barbe V, Daron J, et al. Structural and functional partitioning of bread wheat chromosome 3B. Science. 2014;345:1249721.

16. Brenchley R, Spannagl M, Pfeifer M, Barker GLA, D'Amore R, Allen AM, et al. Analysis of the bread wheat genome using whole-genome shotgun sequencing. Nature. 2012;491:705–10.

17. International Wheat Genome Sequencing Consortium (IWGSC). A chromosome-based draft sequence of the hexaploid bread wheat (Triticum aestivum) genome. Science. 2014;345:1251788.

18. Clavijo BJ, Venturini L, Schudoma C, Accinelli GG, Kaithakottil G, Wright J, et al. An improved assembly and annotation of the allohexaploid wheat genome identifies complete families of agronomic genes and provides genomic evidence for chromosomal translocations. Genome Res. 2017;27:885–96.

19. Avni R, Nave M, Barad O, Baruch K, Twardziok SO, Gundlach H, et al. Wild emmer genome architecture and diversity elucidate wheat evolution and domestication. Science. 2017;357:93–7.

20. Jiao Y, Peluso P, Shi J, Liang T, Stitzer MC, Wang B, et al. Improved maize reference genome with single-molecule technologies. Nature. 2017;546:524–7.

21. Zimin AV, Puiu D, Luo M-C, Zhu T, Koren S, Marçais G, et al. Hybrid assembly of the large and highly repetitive genome of Aegilops tauschii, a progenitor of bread wheat, with the MaSuRCA mega-reads algorithm. Genome Res. 2017;27:787–92.

22. Zimin AV, Puiu D, Hall R, Kingan S, Clavijo BJ, Salzberg SL. The first near-complete assembly of the hexaploid bread wheat genome, Triticum aestivum. Gigascience. 2017;6:1–7.

23. Ma J, Stiller J, Berkman PJ, Wei Y, Rogers J, Feuillet C, et al. Sequence-based analysis of translocations and inversions in bread wheat (Triticum aestivum L.). PLoS One. 2013;8:e79329.

24. Tuzun E, Sharp AJ, Bailey JA, Kaul R, Morrison VA, Pertz LM, et al. Fine-scale structural variation of the human genome. Nat Genet. 2005;37:727–32.

25. Kidd JM, Cooper GM, Donahue WF, Hayden HS, Sampas N, Graves T, et al. Mapping and sequencing of structural variation from eight human genomes. Nature. 2008;453:56–64.

26. Williams LJS, Tabbaa DG, Li N, Berlin AM, Shea TP, Maccallum I, et al. Paired-end sequencing of Fosmid libraries by Illumina. Genome Res. 2012;22:2241–9.

27. Bovee D, Zhou Y, Haugen E, Wu Z, Hayden HS, Gillett W, et al. Closing gaps in the human genome with fosmid resources generated from multiple individuals. Nat Genet. 2008;40:96–101.

28. Jarvis DE, Ho YS, Lightfoot DJ, Schmöckel SM, Li B, Borm TJA, et al. The genome of Chenopodium quinoa. Nature. 2017;542:307–12.

29. Hulse-Kemp AM, Maheshwari S, Stoffel K, Hill TA, Jaffe D, Williams SR, Weisenfeld N, Ramakrishnan S, Kumar V, Shah P, Schatz MC, Church DM, Van Deynze A. Reference quality assembly of the 3.5-Gb genome of Capsicum annuum from a single linked-read library. Hortic Res. 2018 Jan 12;5:4. doi: 10.1038/s41438-017-0011-0.

30. Schmidt MH-W, Vogel A, Denton AK, Istace B, Wormit A, van de Geest H, et al. De Novo Assembly of a New Solanum pennellii Accession Using Nanopore Sequencing. Plant Cell. 2017;29:2336–48.

31. Magoč T, Salzberg SL. FLASH: fast length adjustment of short reads to improve genome assemblies. Bioinformatics. 2011;27:2957–63.

32. Martin M. Cutadapt removes adapter sequences from high-throughput sequencing reads. EMBnet.journal. 2011;17:10.

33. Bolger AM, Lohse M, Usadel B. Trimmomatic: a flexible trimmer for Illumina sequence data. Bioinformatics. 2014;30:2114–20.

34. Andrews S, FastQC: a quality control tool for high throughput sequence data. 2010; https;//www.bioinformatics.babraham.ac.uk/projects/fastqc/

35. Langmead B, Salzberg SL. Fast gapped-read alignment with Bowtie 2. Nat Methods. 2012;9:357–9.

36. Picard**.** http://broadinstitute.github.io/picard**.** Accessed 1st March 2018

37. Lu, FH; McKenzie, N; Kettleborough, G; Heavens, D; Clark, MD; Bevan, MW (2018): Supporting data for "Independent assessment and improvement of wheat genome assemblies using Fosill jumping libraries." GigaScience Database. http://dx.doi.org/10.5524/100446

Figure Legends

Figure 1. Determination of Fosill mate-pair distance distributions on chromosome 3B.

A. 576 M quality controlled paired-end sequences were mapped to the chromosome 3B pseudomolecule. 588,268 read pairs were mapped and the insert sizes calculated. The mean insert size was 37,725 Kb.

B. Fosill mate-pairs were mapped in 100 Kb bins along chromosome 3B to assess the depth and evenness of coverage. Coverage was generally even across the entire chromosome, with approximately 30 very high copy peaks that are probably due to Fosill mate-pairs from highly related 40 Kb+ regions from across the genome. Most mate-pairs mapped to a depth of ″5 and were used for subsequent analyses.

Figure 2. Using Fosill mate-pair matches to identify discrepancies in wheat chromosome and genome assemblies.

A. The schematic describes different classes of matches of Fosill mate-pair sequences to wheat chromosome and genome assemblies. Consistent assemblies matched a span of ≥5 mate-pairs in a sliding 10 Kb “driver” window that matched their mate in a 20 Kb “follower” window at a distance of 37 Kb +/- sd in the correct orientation. Where mate-pairs spanned more than 50 Kb (approximately 3 sd) this was construed to be due to an aberrant insertion in the underlying assembly. Spans <25 Kb (approximately 3 sd) were construed to be due to an aberrant deletion in the assembly. Mis-orientations of the mate-pairs indicated a mis-oriented

assembly, and no span a mis-join in the assembly. New joins were also identified. Drawing not to scale.

B. An example of a mis-join of the BAC-based assembly of chromosome 3B. Two scaffolds, v443_0362 and v443_0787, were originally assembled at opposite ends of chromosome 3B 730 Mb apart. Matches to Fosills indicated that these two scaffolds could be re-assembled together with v443_0362 in the opposite orientation. The Mummer plot shows that this join is supported by TGACv1 scaffold_220633_3B. Drawing not to scale.

C. An example of an aberrant deletion in TGACv1 scaffold 220602 on chromosome 3B. Assembly missed a duplicate copy of a 12 Kb repeat (represented by an arrow) that was identified as a discrepancy in Fosill mate-pair matches. Comparison to a Triticum 3.0 scaffold identifies the predicted missing copy of the repeat. Drawing not to scale.

D. An example of an aberrant insertion in TGAv1 scaffold 591781 on chromosome 7BS detected by Fosill mate-pair matches of >50 Kb. Comparison to the Triticum 3.0 assembly of the same regions identifies the mis-assembled insertion. Drawing not to scale.

Figure 3. Increasing assembly contiguity using Fosill matches.

A. Fosill mate-pair reads were used to link scaffolds of TGACv1 Illumina assemblies from chromosome 3B. The distribution of scaffold lengths and the number of scaffolds in each size range is shown before (dark bars) and after (grey bars) Fosill scaffolding. The numbers

of smaller scaffolds are reduced, and the numbers of larger scaffolds are increased, by Fosill scaffolding, showing successful further assembly.

B. Fosill mate-pair reads were used to link scaffolds of BAC-based assemblies of chromosome 3DL. The distribution of scaffold lengths and the number of scaffolds in each size range is shown before (dark bars) and after (grey bars) Fosill scaffolding. The numbers of smaller scaffolds are reduced, and the numbers of larger scaffolds are increased, by Fosill scaffolding, showing successful further assembly.

Figure 4. Fosill-mediated scaffolding of TGACv1 Illumina assemblies of the wheat genome. The 21 chromosomes are shown with their scaffold N50 values before (black bars) and after (grey bars) Fosill-mediated scaffolding.

Table 1

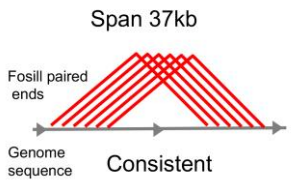	Mean Fosill Insert Size bp	Std Dev	Assembly Size Mb	Scaffold N50 Kb	Total Scaffolds	Consistent Scaffolds	Consistent bases (% of total)
3B BAC Assembly	37,683	11,421	832	892	2,808	1859 (66%)	85.7%
3B TGAC v1 Assembly	37,177	4,608	789	116	29,000	17,730 (61%)	80.5%
3B Triticum 3.0	37,303	3,892	782	372	3,750	3,518 (92%)	91.0%
3B DenovoMAGIC2	37,661	3,968	841	6,373	271	271(100%)	99.6%
3DL BAC Assembly	37,254	5,160	453	154	23,433	4,040 (17%)	57.2%
3DL Triticum 3.0	37,247	3,887	409	279	2,703	2,691 (99.5%)	86.2%
All TGAC v1 (500Kb)	37,489	4,549	943	-	159	159 (100%)	98.9%

A. Summary of Fosill mate-pair alignments to different publically-available assemblies of chromosome 3B and 3DL, and the TGACv1 whole genome assembly. The consistency of mapping is shown according to the number of assemblies with consistent matches, and the percentage of bases included in consistent matches to Fosill mate-pairs.

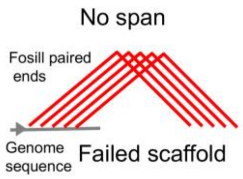	Scaffolding failures	Assemblies involved	Bases Involved Mb
3B BAC Assembly	642	520	2.7
3B TGAC v1 Assembly	314	314	3.6
3B Triticum 3.0	517	499	11.4
3B DenovoMAGIC2	4	1	0.034
3DL BAC Assembly	536	491	17.23
3DL Triticum 3.0	444	442	9.7
All TGAC v1 (500Kb)	11	11	0.054

B. Potential failed scaffolding

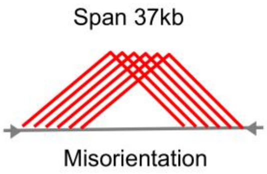	Mis-orientation errors	Assemblies involved	Bases Involved Mb
3B BAC Assembly	92	78	2.7
3B TGAC v1 Assembly	6	6	0.094
3B Triticum 3.0	1	1	0.049
3B DenovoMAGIC2	21	1	1.06
3DL BAC Assembly	8	8	0.214
3DL Triticum 3.0	0	0	0
All TGAC v1 (500Kb)	1	1	0.007

C. Potential mis-orientations in scaffolds

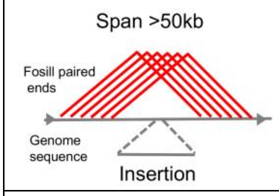	Insertion errors	Assemblies involved	Bases Involved Mb
3B BAC Assembly	255	177	8.06
3B TGAC v1 Assembly	31	27	0.712
3B Triticum 3.0	88	66	1.7
3B DenovoMAGIC2	30	1	0.358
3DL BAC Assembly	78	63	2.225
3DL Triticum 3.0	18	17	0.396
All TGAC v1 (500Kb)	4	4	0.163

D. Potential erroneous insertions in scaffolds

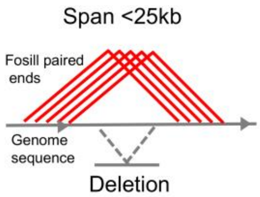	Deletion errors	Assemblies involved	Bases Involved Mb
3B BAC Assembly	626	381	15.97
3B TGAC v1 Assembly	129	116	2.58
3B Triticum 3.0	108	89	2.17
3B DenovoMAGIC2	72	1	0.366
3DL BAC	58	53	1.09
3DL Triticum 3.0	41	36	0.758
All TGAC v1 (500Kb)	13	9	0.307

E. Potential erroneous deletions in scaffolds

 Click here to download Table Table 2.docx

Table 2.

267 new links	Strand	Validated by TGACv1
37 links ≤40kb on pseudomolecule	20 correct strands	12
	17 reverse strands	7
147 links >40kb on pseudomolecule	73 correct strands	31
	74 reverse strands	38
83 links between scaffolds not assigned to the pseudomolecule	-	36

A. Summary of new links made between BAC scaffolds on chromosome 3B.

	**chr3B TGACv1**		**chr3DL BACs**	
	**Before**	**After**	**Before**	**After**
Bases (bp)	789,970,040	846,817,359	452,947,627	463,673,958
Assemblies	29,090	22,014	23,433	21,985
Number scaffolds >n50	2,020	644	790	721
Min	500	500	501	501
n10	293,318	947,439	463,110	933,142
n50	116,546	398,569	154,985	286,993
Max	739,616	2,867,878	1,240,092	1,942,124

2B. Summary of changes in assemblies of chromosome 3B TGACv1 and chromosome 3DL BAC assemblies.

267 new links	Strand	Validated by TGACv1
37 links ≤40kb on pseudomolecule	20 correct strand	12
	17 reverse strand	7
147 links >40kb on pseudomolecule	73 correct strand	31
	74 reverse strand	38
83 links between scaffolds not assigned to the pseudomolecule	-	36

Table 3A. Summary of new links made between BAC scaffolds on chromosome 3B.

	**chr3B TGACv1**		**chr3DL BACs**	
	**Before**	**After**	**Before**	**After**
**Bases**	789,970,040	846,817,359	452,947,627	463,673,958
**Assemblies**	29,090	22,014	23,433	21,985
**Number scaffolds >n50**	2,020	644	790	721
**Min**	500	500	501	501
**n10**	293,318	947,439	463,110	933,142
**n50**	116,546	398,569	154,985	286,993
**Max**	739,616	2,867,878	1,240,092	1,942,124

Table 3B. Summary of changes in assemblies of chromosome 3B TGACv1 and chromosome 3DL BAC assemblies.

Click here to download Figure Figure 1.pdf

Figure 1.

A

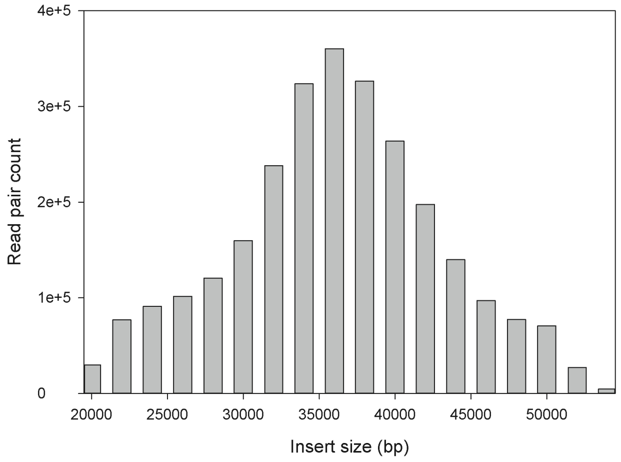

B

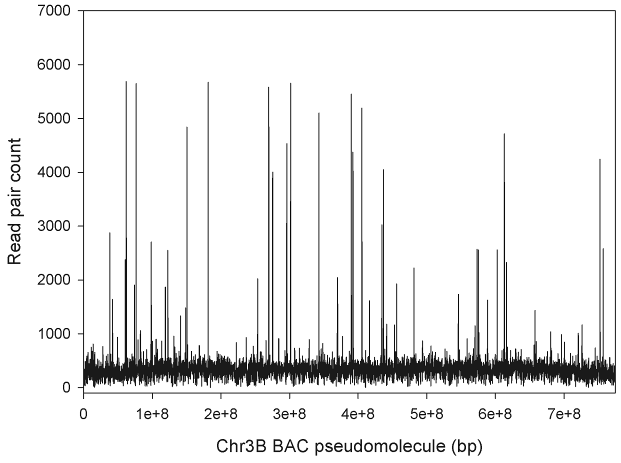

Figure 2 Click here to download Figure Figure 2.pdf

Figure 2.

2A

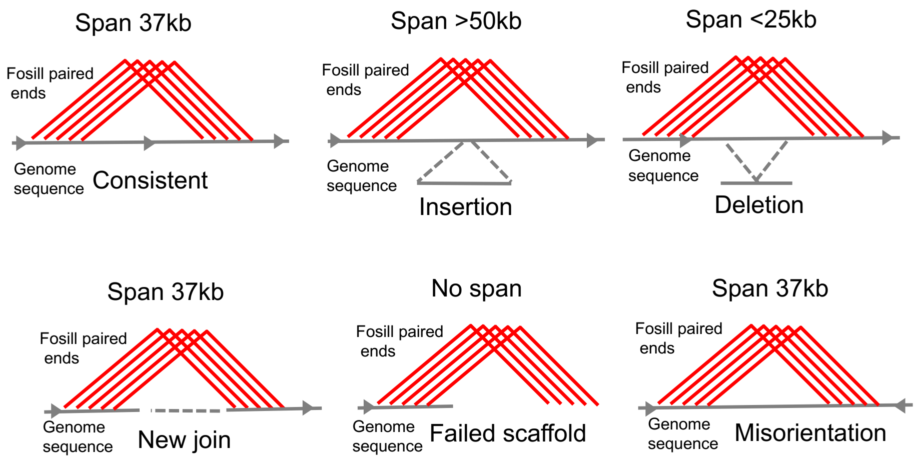

2B

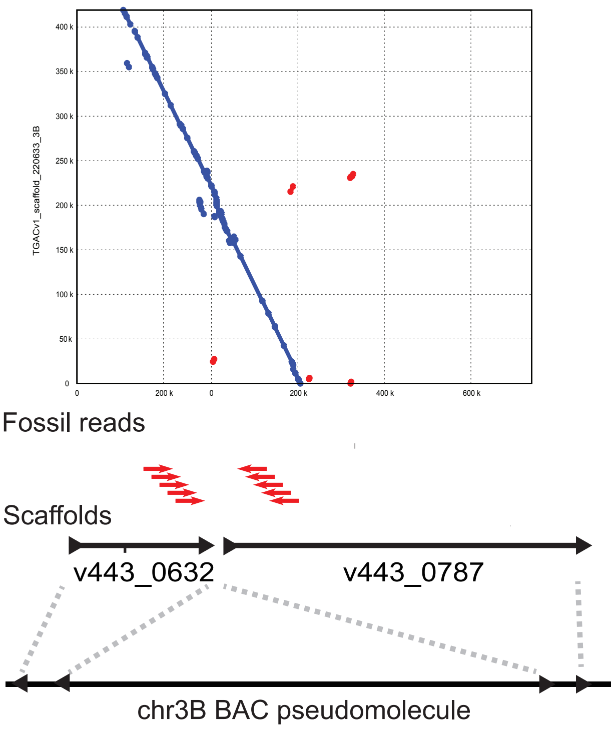

2C

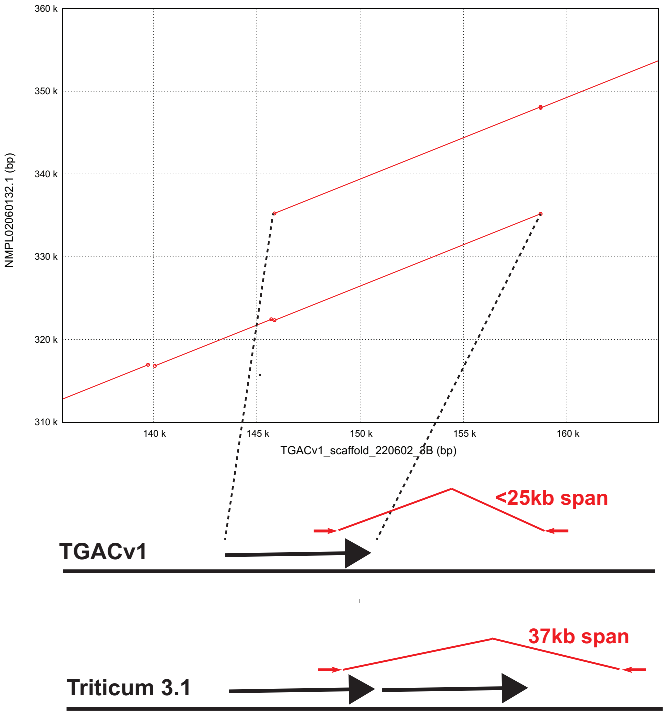
360 k
350 k
340 k
330 k
320 k
310 k
NMPL02060132.1 (bp)
140 k
145 k
150 k
155 k
160 k
TGACv1_scaffold_220602_3B (bp)
<25kb span
TGACv1
37kb span
Triticum 3.1

2D

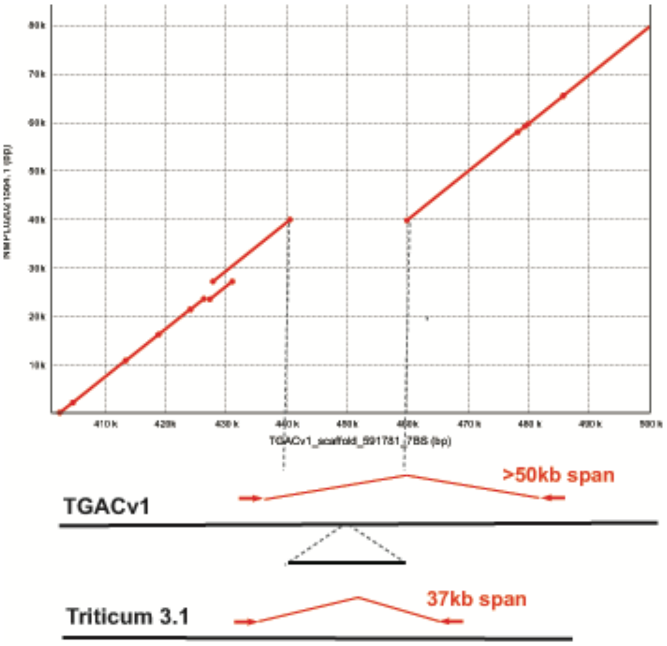
>50kb span
TGACv1
37kb span
Triticum 3.1

Figure 3

A

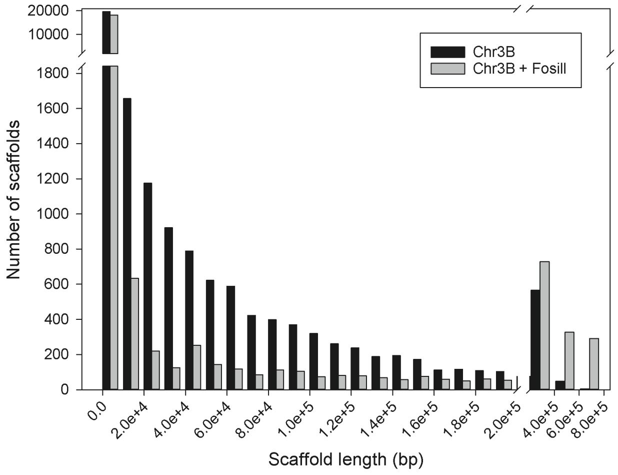
20000
10000
1800
1600
1400
1200
1000
800
600
400
200
0
Number of scaffolds
Chr3B
Chr3B + Fosill
0.0
2.0e+4
4.0e+4
6.0e+4
8.0e+4
1.0e+5
1.2e+5
1.4e+5
1.6e+5
1.8e+5
2.0e+5
4.0e+5
6.0e+5
8.0e+5
Scaffold length (bp)

B

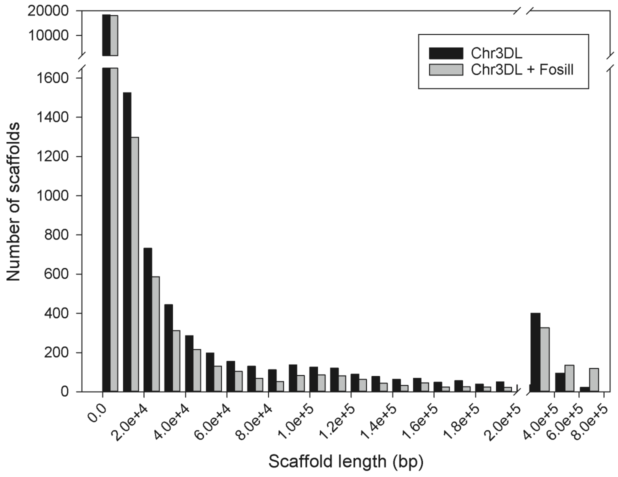
20000
10000
1600
1400
1200
1000
800
600
400
200
0
Number of scaffolds
Chr3DL
Chr3DL + Fosill
0.0
2.0e+4
4.0e+4
6.0e+4
8.0e+4
1.0e+5
1.2e+5
1.4e+5
1.6e+5
1.8e+5
2.0e+5
4.0e+5
6.0e+5
8.0e+5
Scaffold length (bp)

Figure 4.

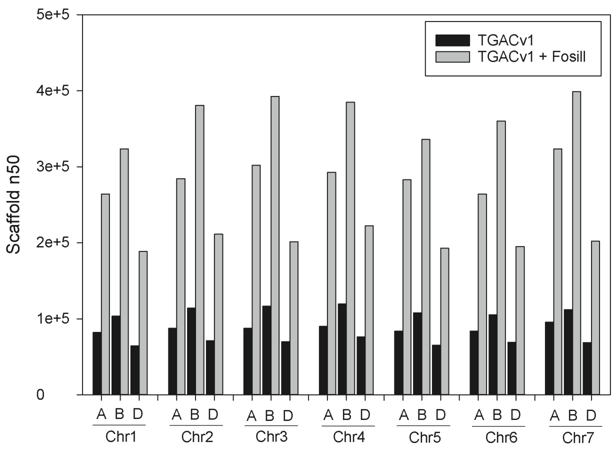
5e+5
4e+5
3e+5
2e+5
1e+5
0
Scaffold n50
TGACv1
TGACv1 + Fosill
A B D
Chr1
Chr2
Chr3
Chr4
Chr5
Chr6
Chr7

Additional File 1

Click here to access/download

Supplementary Material

Additional File 1.docx

Additional File 2

Click here to access/download
Supplementary Material
Additional File 2.docx

Additional File 3

Click here to access/download
Supplementary Material
Additional File 3.docx

Additional File 4

Click here to access/download
Supplementary Material
Additional File 4.xls

Additional File 5

Click here to access/download
Supplementary Material
Additional File 5.xls

Additional File 6

Click here to access/download
Supplementary Material
Additional File 6 new.docx

Click here to access/download

Supplementary Material

Additional File 7. TGACv2 scaffolds.xlsx
