# Supplementary material for: Independent assessment and improvement of wheat genome sequence assemblies using Fosill jumping libraries
Source: Gigascience. 2018 May 11;7(5):giy053. doi: 10.1093/gigascience/giy053 (PMC5967450; doi:10.1093/gigascience/giy053)
Supplement: Supplemental material [file giy053_supp.zip › Additional File 1.docx]

**Additional File 1**

**Genome assembly simulation**

Before adopting Fosill jumping libraries for analysing and improving wheat genome assemblies, we simulated assembly processes using Fosill mate-pair reads on three of the largest scaffolds of the BAC-based assembly of chromosome 3B [1]. Simulations used Next-Generation illumina SIMulation PipeLinE (NGSimple, <https://github.com/lufuhao/NGSimple)> to assess various parameters, including library types, fragment sizes, read length, and sequencing depth. Simulated reads were generated by the Mason program [2] and then trimmed by Trimmomatic version 0.32 [3]. Quality control was applied to these datasets before and after, in order to confirm the removal of the low quality and low complexity bases in reads. Velvet v1.2.10 [4] was used to assemble these reads from different parameter settings in one run. And finally, MUMmer v3.23 [5] was used to map the assembled contigs back to its original scaffold to evaluate the quality of the faux assemblies. Results for a single representative scaffold are shown below.

| Chromosome 3B scaffolds used for simulation. These were downloaded from <https://urgi.versailles.inra.fr/download/wheat/3B/ta3bAllScaffoldsV443.genom.fa.gz> |
| --- |

| **Scaffold ID** | **Length** |
| --- | --- |
| v443_0936 | 4,169,843 bp |
| v443_0903 | 3,662,090 bp |
| v443_0899 | 3,466,457 bp |

Simulation parameter settings

| **Parameters** | **Settings** | | |
| --- | --- | --- | --- |
| Library types | Paired end | Mate pair | Long mate pair |
| Fragment sizes (bp) | 600 | 3-10 kb | 20K, 40 kb |
| Read lengths | 100, 120, 150, 175, 200, 250 bp | | |
| Sequencing depths | 50X | 2-10X | 0.1-2X |

**1.1 Optimizing mate-pair fragment length**

Simulation settings for mate-pair library fragment sizes

|  | Insert size | Coverage | Read length |
| --- | --- | --- | --- |
| Paired-end | 600 | 50x | 250 bp |
| Mate pair | 3,000-10,000 | 10x | 250 bp |
| Long mate pair | 20K, 40K | 2x | 250 bp |


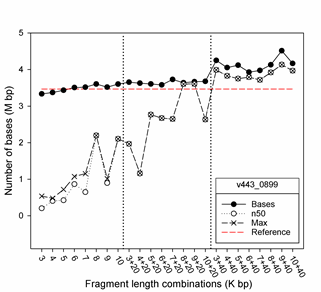


**Fig.1** Summary of the *de novo* assemblies based on scaffold v443_0899


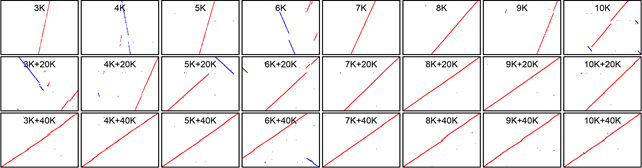


**Fig.2** Plot of longest contig against its reference v443_0899

**1.2 Optimizing mate-pair sequence read length**

**Table 2.** Simulation settings for sequence read length

|  | Insert size | Coverage | Read length |
| --- | --- | --- | --- |
| Paired-end | 600 | 30x | 100-300 bp |
| Mate paired | 7,000 | 10x | 100-300 bp |
| Long mate paired | 40,000 | 2x | 100-300 bp |


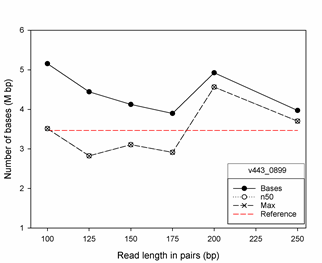


**Fig.3** Summary of the *de novo* assemblies based on v443_0899


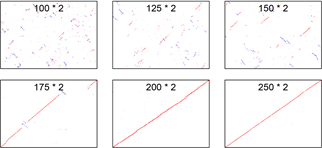


**Fig.4** Plot of longest contig against its reference v443_0899

**1.3 Optimizing long mate-pair coverage**

**Table 3.** Simulation Setting for coverage by long mate-pair libraries. “0x” means no 40 Kb mate pairs were used in the simulation.

|  | Insert size | Coverage | Read length |
| --- | --- | --- | --- |
| Paired-end | 600 | 50x | 250 bp |
| Mate paired | 7,000 | 10x | 250 bp |
| Long mate paired | 40,000 | 0-2x | 250 bp |


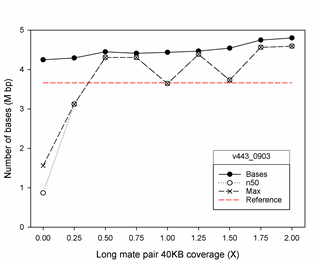


**Fig.5** Summary of the *de novo* assemblies based on v443_0903


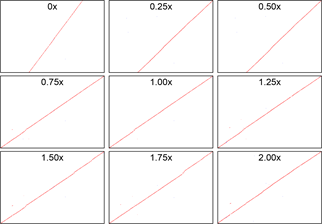


**Fig.6** Plot of longest contig against its reference v443_0903

**Table 4.** Simulation Setting for coverage by mate-pair libraries

|  | Insert size | Coverage | Read length |
| --- | --- | --- | --- |
| Paired-end | 600 | 50x | 250 bp |
| Mate paired | 7,000 | 2-10x | 250 bp |
| Long mate paired | 40,000 | 1x | 250 bp |


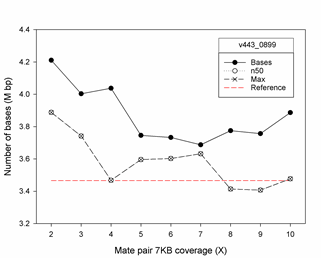


**Fig.7** Summary of the *de novo* assemblies based on v443_0899


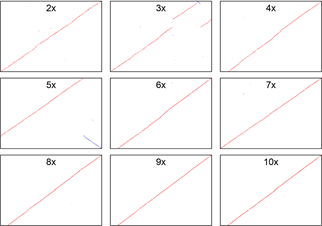


**Fig.8** Plot of longest contig against its reference v443_0899

**References**

1. Choulet F, Alberti A, Theil S, Glover N, Barbe V, Daron J, et al. Structural and functional partitioning of bread wheat chromosome 3B. Science. 2014;345:1249721.

2. Holtgrewe M. 2010. Mason - a read simulator for second generation sequencing data. Diploma Thesis. Repository: Freie Universität Berlin, Math Dept

3. Bolger AM, Lohse M, Usadel B. Trimmomatic: a flexible trimmer for Illumina sequence data. Bioinformatics. 2014;30:2114–20.

4. Zerbino DR, Birney E. Velvet: Algorithms for *de novo* short read assembly using de Bruijn graphs. Genome Research. 2008;18:821–9.

5. Kurtz S, Phillippy A, Delcher AL, Smoot M, Shumway M, Antonescu C, et al. Versatile and open software for comparing large genomes. Genome Biology. 2004;5:R12.
