# Supplementary material for: Independent assessment and improvement of wheat genome sequence assemblies using Fosill jumping libraries
Source: Gigascience. 2018 May 11;7(5):giy053. doi: 10.1093/gigascience/giy053 (PMC5967450; doi:10.1093/gigascience/giy053)
Supplement: Supplemental material [file giy053_supp.zip › Additional File 2.docx]

**Additional File 2**

**Fosill library production**

The pFosill 4 cloning vector was used in this study was kindly provided by Louise Williams (Broad Institute, Cambridge, MA, USA). The construction, preparation and downstream steps for non-size-selected DNA fragments were carried out as described [1]. Only differences in the methods are described here.

A single-seed-descent line of *Triticum aestivum* Chinese Spring (CS42) was used for high molecular weight DNA extraction as described [2]. 30ug of Genomic DNA was sheared to approximately 40kb average fragment size by HydroShear (Digilab, Marlborough, MA, USA) using the Large Shearing Assembly set at speed code 40 for 20 cycles. Sheared DNA was assessed by Pulse Field Gel Electrophoresis. 0.5-1ug of sheared and non-sheared DNA was run on BioRad CHEF DRII. 120 degrees, 6V/cm, 1 to 10 second ramp switch, 17 hours at 14⁰C. DNA was visualised by staining gel with Ethidium Bromide (Figure 1).

10ug batches of sheared DNA was end- repaired in 175ul reactions containing 1 X T4 ligase buffer, 0.25mM dNTPs, 15 units T4 DNA polymerase, 50 units T4 polynucleotide Kinase, and 5 units Klenow fragment (all NEB) for 30 mins at 20⁰C. TE was added to the DNA up to 400ul, which was then cleaned and concentrated through Amicon Ultra 0.5ml 100k concentrator (Millipore) at 2000g to approximately 30ul. Recovered DNA was measured on the Qubit fluorometer (Invitrogen) using Quant-iT dsDNA BR kit.

t-b index linker A: GATCTCTACCAGG and t-b index linker B: CCTGGTAGAG were annealed and multiple ligations were set up using 500ng end repaired genomic DNA and 200-fold molar excess of annealed linker. DNA was pooled and between 5 (500ul) to 20 (2000ul) ligations were cleaned and concentrated with one Amicon column.

Multiple 10ul ligations were set up containing 250ng linkered DNA and 500ng cut and dephosphorylated pFossill 4 vector. 10ul ligation was packaged with 2 successive 50ul MaxPlax λ packaging extract (Epicentre) for 90 mins at 30⁰C. 1850ul Phage dilution buffer and 140ul DMSO were added (2100ul total volume) The libraries were titered and stored at -80⁰C. λ –competent GC10 (Sigma) was used for processing packaged libraries into fosmid DNA. A proportion of 1.5ml λ packaged sample to 40 ml of cells was found to give best transformation efficiency. Cultures were grown overnight at 30^o^C in LB.

Fosmid DNA was isolated from LB culture using Qiagen’s Plamid Maxi Purification Kit. 20ml of Solutions P1, P2 and P3 were used and the supernatant was transferred to a new tube through a layer of Miracloth prior to addition to the Maxi column. Fosmid DNA was eluted with 500ul TE and quantified using a Qubit fluorometer.

**Conversation of Fosmids into Fossills.**

Pools of approximately 2.5 million independent Fosill clones were collected (see Table 1) and 10ug of DNA from each pool was processed, with the following modifications. 900ng was nicked with Nb.BbvCI for between 55-60 mins, before S1 nuclease treatment. 300ng of DNA was re-circularised in 650ul containing 1x T4 ligase buffer and 8000 units of T4 ligase (NEB) at 16⁰C for 16 hours. Products were purified using a Qiagen PCR cleanup kit. Columns were washed twice with 750ul wash buffer and eluted with 55ul TE.

A trial PCR was used to determine minimal amplification required for Illumina template preparation. 2ul of re-circularised DNA was amplified in 25ul total volume of 1x Phusion HF master mix and 0.5uM PCR primers:

SBS3: 5’AATGATACGGCGACCACCGAGATCTACACTCTTTTCCCTACACGACGC  3’

SBS12: 5’AAGCAGAAGACGGCATACGAGATGATCGATCGTGACTGGAGTTCAGACGTGTGC 3’

Cycling parameters were 98⁰C for 3 mins, 16 and 18 cycles respectively of 98⁰C for 15 secs, 65⁰C for 30 secs, 72⁰C for 30 secs and a final extension at 72⁰C for 7 mins. PCR products were analysed with a MultiNA Bioanalyser (Shimadzu) using DNA 12000 reagent kit in on-chip mode. An intensity measurement of 5-8MV, which equated to approximately 7.5ng/ul to 12ng/ul for the 700-950 bp peak, was optimal. Following analysis of MultiNA data to determine minimal cycling conditions for each pool, Super-Pools of approximately 10 million independent Fosill clones were selected from the pools and minimal cycle number calculated for each pool to give sufficient material for sequencing. A total of 24 50ul PCR reactions each containing 4ul of Fossill DNA for each Super-Pool. Cycling parameters, primers and primer concentration were same as for trial PCR (except for varied cycle numbers). PCR products from Super-Pools were combined (1200ul) and purified with AMPure XP beads and eluted with 40ul of TE. 4ul of sample was used for MultiNA analysis to confirm size range and quantity. 30ul of sample was size-selected on 1.5% agarose cassette with R2 marker using Sage Science BluePippin (Beverly, MA, USA) set to collect fragments between 650-1000bp. Successful size selection was confirmed using TapeStation size measurement, and DNA was purified with AMPure XP beads and eluted in 25ul TE. Sequencing was performed using 2x250bp pair-end sequencing chemistry on an Illumina HiSeq 2500 sequencer.

Physical coverage of the wheat genome was calculated as: genome size = 15 x 10^9^ bp / clone size = 38 x 10^3^ bp = 0.39M fosill clones = 1x physical coverage.

Sequence read coverage C of the wheat genome was calculated as C = Number of reads x Read Length / Genome Size (500 bp / 15 x 10^9^ bp). 54 x 10^6^ Fosill clones = 1.8 sequence coverage.


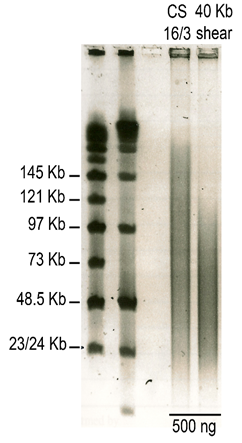


**Figure 1. PFGE analysis of sheared DNA for Fosill vector cloning**

| Library | Titre (M) | Platform | Number of Reads | PCR redundancy |
| --- | --- | --- | --- | --- |
| Lib17562 | 0.90 | MiSeq | 7,283,029 | 6 |
| Lib18185 | 5.50 | HiSeq | 51,668,481 | 9.74 |
| Lib18186 | 5.80 | HiSeq | 55,092,287 | 9.35 |
| Lib19454 | 10.09 | HiSeq | 124,755,368 | 11.68 |
| Lib19455 | 9.99 | HiSeq | 117,879,434 | 9.14 |
| Lib19456 | 11.59 | HiSeq | 111,113,269 | 6.94 |
| Lib19457 | 11.64 | HiSeq | 108,714,323 | 6.89 |
| Total | 55.51 | - | 576,506,191 | 8.51 average |

**Table 1. Summary of Fosill libraries and paired-end reads generated**

The sequences of these libraries can be downloaded from the European Nucleotide Archive (ENA) study section PRJEB23322

<https://www.ebi.ac.uk/ena/data/search?query=PRJEB23322>

**References**

1. Williams LJS, Tabbaa DG, Li N, Berlin AM, Shea TP, MacCallum I, et al. Paired-end sequencing of Fosmid libraries by Illumina. Genome Research. 2012;22:2241–9.

2. Clavijo BJ, Venturini L, Schudoma C, Accinelli GG, Kaithakottil G, Wright J, et al. An improved assembly and annotation of the allohexaploid wheat genome identifies complete families of agronomic genes and provides genomic evidence for chromosomal translocations. Genome Research. 2017;27:885–96.
