# Supplementary material for: Independent assessment and improvement of wheat genome sequence assemblies using Fosill jumping libraries
Source: Gigascience. 2018 May 11;7(5):giy053. doi: 10.1093/gigascience/giy053 (PMC5967450; doi:10.1093/gigascience/giy053)
Supplement: Supplemental material [file giy053_supp.zip › Additional File 3.docx]

**Additional File 3.**

**Fosill mate-pair mapping**

Joinable read pairs from Illumina Miseq or HiSeq sequencing were removed using FLASH v1.2.11 [1]. Ligation adaptors in reads were trimmed off using CutAdapt v1.6 [2]. Sequencing primer sequences and low- quality sequences in reads were removed using Trimmomatic v0.32 [3]. Resulting reads were then evaluated using FastQC v1.2.11 [4].

| Trimmed Fosill mate-pair reads were filtered using the ReadCleaner4Scaffolding pipeline (<https://github.com/lufuhao/ReadCleaner4Scaffolding>). Both mates of each pair were mapped  to chr3B BAC scaffolds  (<https://urgi.versailles.inra.fr/download/wheat/3B/ta3bAllScaffoldsV443.genom.fa.gz> ) |  |  |  |  |  |  |  |
| --- | --- | --- | --- | --- | --- | --- | --- |

using bowtie v1.0.1 [5]. Picard MarkDuplicates (v1.108, <http://broadinstitute.github.io/picard>) was then used to remove duplicates as single reads. A read depth threshold was determined to remove any highly repetitive reads by plotting the summary of output from samtools depth, and all the reads mapped to those regions with depth >5 were not considered for scaffolding. Reads mapping to multiple positions, whose mates were not mapped, or were in the wrong orientation, were removed. A window sizing method was used to map mate-pairs to genomic regions. A group of five neighbouring mate reads within a “driver” window of less than 10kb were linked by the average 37.7kb insertion size +/-sd to a “follower” window of 20kb. Figure 1 shows that nearly all mate-pairs map to chromosome 3B using these criteria. Reads mapping within these window criteria were used to define regions of chromosomes that were consistent with the average insertion size, or had inconsistent matches.

To generate coordinates of each scaffold on the 3B pseudomolecule, BAC scaffolds were mapped to the pseudomolecule and plotted using SyntenyDraw (available on <https://github.com/lufuhao/SyntenyPlot>). These coordinates were compared with our evidence from ReadCleaner4Scaffolding pipeline. To validate mapping, mate-pairs were mapped TGAC v1 chr3B contigs.

Filtered Fosill mate-pair reads were mapped to the TGACv1 whole genome assembly of Chinese Spring 42 as described above, using SSPACE v3.0 to join scaffolds with five fossils links.

X

Y

Driver window

Follower window


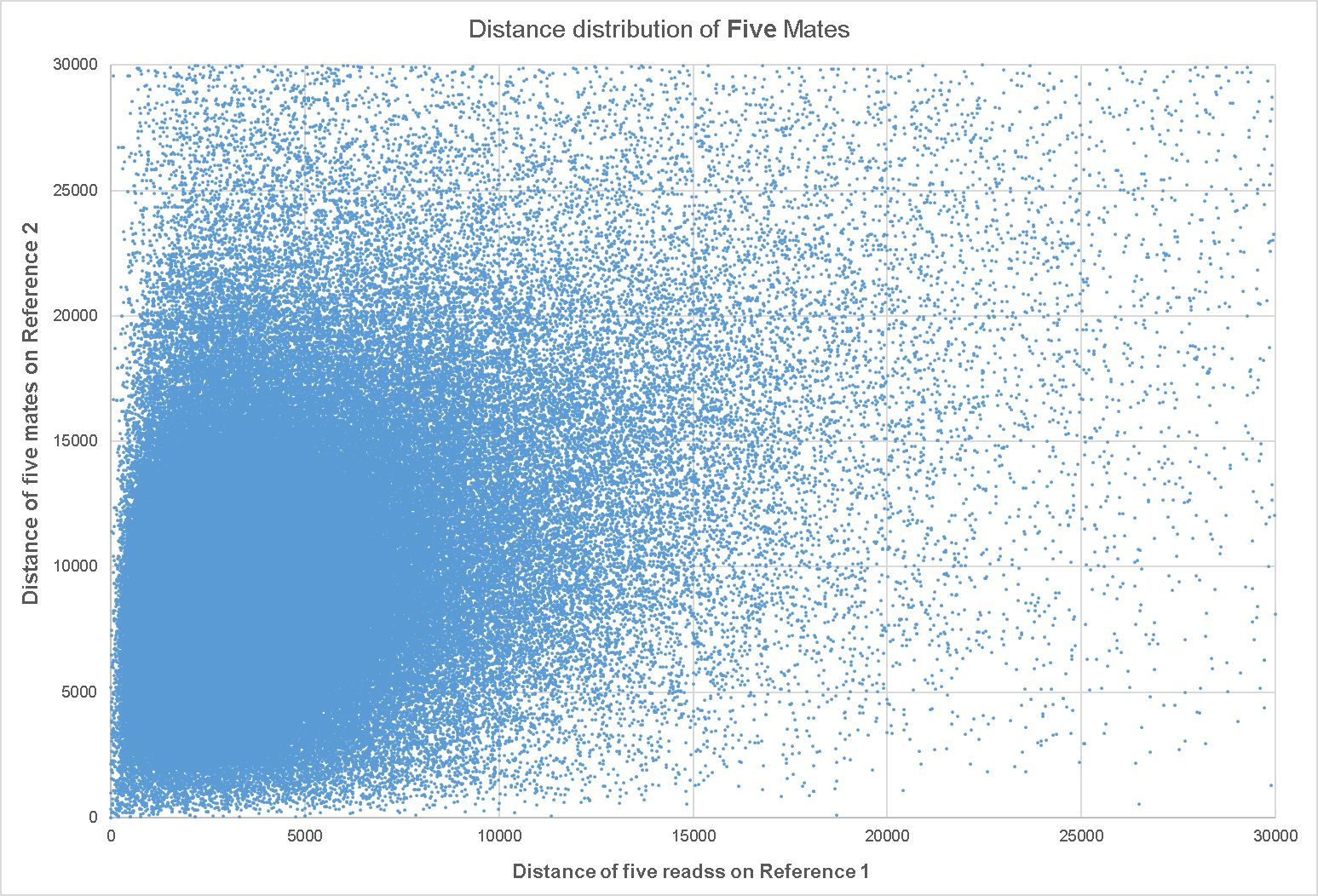


Driver window size (bp)

Follower window size (bp)

**Figure 1. Mapping Fosill mate pairs to genomic scaffolds**

The distribution of mate-pair links of five adjacent Fosill mate-pairs in different sized windows to their corresponding mate-pair read at 37.7 kb +/-sd (standard deviation) was mapped on chromosome 3B BACs. The vast majority of mate-pairs in a 10kb window were found in 20kb windows at the correct 37.7kb distance. These window sizes were used to map Fosill mate-pairs to sequence scaffolds.

**References**

1. Martin M. Cutadapt removes adapter sequences from high-throughput sequencing reads. EMBnetjournal 2017;17:10-12.

2. Magoc T, Salzberg SL. FLASH: fast length adjustment of short reads to improve genome assemblies. Bioinformatics. 2011;27:2957–63.

3. Bolger AM, Lohse M, Usadel B. Trimmomatic: a flexible trimmer for Illumina sequence data. Bioinformatics. 2014;30:2114–20.

4. Andrews S. 2010. FastQC: a quality control tool for high throughput sequence data. [*http://wwwbioinformaticsbabrahamacuk/projects/fastqc/*](http://wwwbioinformaticsbabrahamacuk/projects/fastqc/) doi:citeulike-article-id:11583827.

5. Langmead B, Salzberg SL. Fast gapped-read alignment with Bowtie 2. Nat Meth. 2012;9:357–9.
