# Supplementary material for: Independent assessment and improvement of wheat genome sequence assemblies using Fosill jumping libraries
Source: Gigascience. 2018 May 11;7(5):giy053. doi: 10.1093/gigascience/giy053 (PMC5967450; doi:10.1093/gigascience/giy053)
Supplement: Supplemental material [file giy053_supp.zip › Additional File 6 new.docx]

**Additional File 6.**

**Sequencing Chromosome 3DL BAC minimal tiling path**

**BAC library preparation and sequencing**

The 3DL BAC library was prepared from flow sorted chromosomes [1] at The Institute of Experimental Botany, Olomouc, Czech Republic, and was fingerprinted at CNRGV (Toulouse, France) using SNaPshot-based high information content methods [2]. The raw fingerprint data was processed according to IWGSC guidelines. LTC [3] was used to build the physical map and generate a minimum tiling path (MTP). The final path consisted of 620 fingerprint contigs (FPCs) containing 5 or more BACs. 6338 BACs were selected for sequencing (6252 MTP clones plus 86 bridge clones).

Paired-end and long mate-pair (LMP) libraries were prepared and sequenced to generate PE reads for each BAC and a pool of LMP reads for each 384 well plate of BACs. LMP reads were processed as described in [4]. After standard QC, filtering and de-multiplexing, the reads were ready for assembly.

**BAC assembly and mate-pair preparation**

Reads were aligned to *E. coli* DH10B, wheat chloroplast and mitochondria sequences using Bowtie 2 [5]. Read pairs with one or more reads mapping with 95% identity or above were removed. Reads were also aligned to the pIndigoBAC-5 BAC vector sequence. Read pairs where one or more reads mapped to the middle of the vector sequence were removed while pairs where a read mapped to the end of a vector sequence were kept. This identified vector insert ligation sites. BACs were assembled individually using Abyss [6]. The BAC assemblies had an average insert size of 112886 bp and an average N50 of 25214 bp. In addition to the pooled LMPs, we used the 9 kbp and 12 kbp whole genome wheat LMPs from [4]. These were first filtered for non-3DL reads by alignment to the IWGSC CSS assembly where all 3DL contigs were replaced with our BAC assemblies. Reads were assigned to individual BACs as a side effect of this process.

We then assigned reads from the pooled LMPs to each BAC. A Jellyfish [7] 31-mer hash table was generated from each assembly and these were combined to create a table of 31-mers found in the BACs on each plate. To identify LMP reads matching BACs, the

“sect” function of the Kmer Analysis Toolkit (KAT) [8] v1.0.5 was used to generate a *k*-mer coverage profile of each LMP read in each pool using plate-specific PE *k*-mer hash tables. The plate- specific LMP reads were then classified to individual BACs on that plate using *k*-mers from individual BAC assemblies.

**Chromosome arm assembly**

Before any scaffolding, the BAC assemblies belonging to each FPC were then merged to remove redundancy. This was done first using CD-HIT [9] and then BLAST [10]. Any overlapping sequence at the end of two BAC contigs of at least 98 % identity and 1000 bp in length resulted in the two contigs being merged into one new contig. Following this procedure each FPC had an average size of 460 kbp and an N50 of 17 kbp.

The non-redundant FPCs were then scaffolded using Soapdenovo [11] with the assigned pooled and whole genome LMP reads. This resulted in an average FPC size of 782 kbp and an N50 of 180 kbp. Finally, the FPC sequences were combined and the merging process was run again. This resulted in a total size of 455 Mbp for the whole chromosome arm and an N50 of 145 kbp.

**References**

1. Safar J, Bartos J, Janda J, Bellec A, Kubaláková M, Valárik M, et al. Dissecting large and complex genomes: flow sorting and BAC cloning of individual chromosomes from bread wheat. Plant J. 2004;39:960–8.

2. Luo M-C, Thomas C, You FM, Hsiao J, Ouyang S, Buell CR, et al. High-throughput fingerprinting of bacterial artificial chromosomes using the snapshot labeling kit and sizing of restriction fragments by capillary electrophoresis. Genomics. 2003;82:378–89.

3. Frenkel Z, Paux E, Mester D, Feuillet C, Korol A. LTC: a novel algorithm to improve the efficiency of contig assembly for physical mapping incomplex genomes. BMC Bioinformatics. 2010;11:584.

4. Clavijo BJ, Venturini L, Schudoma C, Accinelli GG, Kaithakottil G, Wright J, et al. An improved assembly and annotation of the allohexaploid wheat genome identifies complete families of agronomic genes and provides genomic evidence for chromosomal translocations. Genome Research. 2017;27:885–96.

5. Langmead B, Salzberg SL. Fast gapped-read alignment with Bowtie 2. Nat Meth. 2012;9:357–9.

6. Simpson JT, Wong K, Jackman SD, Schein JE, Jones SJM, Birol I. ABySS: A parallel assembler for short read sequence data. Genome Research. 2009;19:1117–23.

7. Marçais G, Kingsford C. A fast, lock-free approach for efficient parallel counting of occurrences of k-mers. Bioinformatics. 2011;27:764–70.

8. Mapleson D, Garcia Accinelli G, Kettleborough G, Wright J, Clavijo BJ. KAT: a K-mer analysis toolkit to quality control NGS datasets and genome assemblies. Bioinformatics. 2017;33:574–6.

9. Fu L, Niu B, Zhu Z, Wu S, Li W. CD-HIT: accelerated for clustering the next-generation sequencing data. Bioinformatics. 2012;28:3150–2.

10. S. F. Altschul et al. “Basic local alignment search tool”. Journal of Molecular Biology 215.3 1990;215:403–410.

11. Luo R, Liu B, Xie Y, Li Z, Huang W, Yuan J, et al. SOAPdenovo2: an empirically improved memory-efficient short-read de novo assembler. Gigascience. 2012;1:18.
