# Supplementary material for: Independent assessment and improvement of wheat genome sequence assemblies using Fosill jumping libraries
Source: Gigascience. 2018 May 11;7(5):giy053. doi: 10.1093/gigascience/giy053 (PMC5967450; doi:10.1093/gigascience/giy053)
Supplement: Response_to_Reviewer_Comments_Original_Submission.pdf [file giy053_response_to_reviewer_comments_original_submission.pdf]

Author's Response To Reviewer Comments

Close

Responses to Reviewers' comments GIGA-D-17-00308

Reviewer x

This reviewer makes important points about the comparison of different types of assemblies and about how they should not be approached as "equivalent assemblies". This is important because different assembly types (BAC vs WGS) may have different error types at different scales; eg BAC-based assemblies are based on a minimal tiling path (MTP) of BACS (often supported by other data types such as genetic maps), and the longer- range scale assembly accuracy of such MTPs may be more accurate than those of whole genome shotgun (WGS) assemblies, which are generated de novo with no supporting information.

To address this concern, we have refocussed the Introduction and Discussion to make clear that the Fosill 40kb mate pair mapping approach described in the paper assesses shorter sequence assembly scales and not longer, pseudo-molecule scale, assemblies. The title has also been edited to add the word "sequence" before assemblies in order show that 40 kb long mate-pairs are used to assess the fidelity of sequence contigs and scaffolds on the scale of 100-1000 Kb. This is the contig and scaffold size range of sequence assembly scales from both WGS and BAC-based sequence assemblies- from 100-1000kb. So, we think it is reasonable to compare the different assemblies at this scale. Similar approaches have been taken in assessing different human genome sequence assemblies. It's important to note however that assembly errors at these shorter scales can be propagated into larger-scale errors unless they are detected by eg HiC, linked reads, or optical mapping. Hence it is timely, at this stage of wheat genomics, to assess sequence assembly scale differences using a common approach.

Our primary motivation for assessing the fidelities of sequence assemblies is to help guide the next steps of sequencing technology for wheat, hence the focus on sequence assembly and scaffold accuracy. We did not set out to assess larger-scale fidelity as 40 kb mate pairs are just not suitable for this. We hope the revised manuscript makes our objectives clearer. For example, in the Discussion we suggest that HiC, linked reads and perhaps Nanopore long reads can be used to generate and assess longer scale assemblies.

We believe we have addressed the issues raised and, as Reviewer x comments, the findings described in the paper would "be extremely useful" for guiding assembly strategies.

Reviewer 1

This reviewer states that the work described is a timely contribution to wheat genome assembly work and states that the paper would be suitable for publication subject to specific edits. These have been done as follows:

1. corrected to "avoid"
2. (parameter)- deleted
3. corrected to "were mapped"

4. sd = standard deviation defined
5. corrected to "publicly"
6. Num> n50 now spelt out in Table 3B

Reviewer 3

This reviewer finds the work to be interesting and provides readers with useful information for guiding genome assembly.

Specific comments

A detailed description of how to construct a Fosill library is provided in Additional File 2. This describes steps from DNA preparation through to specific modifications for wheat.

Regarding SMRT reads, the manuscript has been edited again to make sure that SMRT reads formed part of the independent Triticum v 3.0 assembly generated by Alexi Zimin and colleagues. We did not use SMRT reads to assess assemblies. Considering whether Fosill can "exist together" or be used together with Nanopore /PacBio sequences to create improved assemblies, the work described in this manuscript indicates that such long reads need to be very accurate to avoid mis-assembly. In the case of Nanopore sequences, these have, to the best of our knowledge, not yet been used in wheat assemblies. SMRT sequences as used in the Triticum 3.0 assembly must be fully corrected with Illumina sequences before they can be used to create accurate assemblies. Once this is done then Fosill mate-pair reads can be incorporated with accurate long reads in wheat assemblies. In fact, Fossil libraries are very hard to make at sufficient coverage, so it's likely that they will not be generally used in future wheat assemblies.

L90-95
Here in the Background we aim to provide a brief summary of how two large and challenging genomes of pine and wheat have been sequenced. Long reads have been very important for generating good assemblies, therefore their use does need to be described. Hence our desire to assess the fidelity of these different assemblies. The Background section has been re-organised and edited to help clarity.

L138
Physical and sequence coverage calculations are now shown in Additional File 2.

Table 1 has been edited so that all values are to the first decimal point

Additional File 1, Table 3 and Figure 5.

Here 0 x coverage means that the simulation used no 40kb mate-pairs in the simulation. So it does mean "no data". The legend to Table 3 in Additional File 1 has been modified to explain what 0 means.

Close
