# Supplementary material for: Independent assessment and improvement of wheat genome sequence assemblies using Fosill jumping libraries
Source: Gigascience. 2018 May 11;7(5):giy053. doi: 10.1093/gigascience/giy053 (PMC5967450; doi:10.1093/gigascience/giy053)
Supplement: Reviewer_1_Report_(Original_Submission) -- Martin Mascher — 12/27/2017 Reviewed [file giy053_reviewer_1_report_(original_submission).pdf]

Reviewer Report

Title: Independent assessment and improvement of wheat genome sequence assemblies using Fosill jumping libraries.

Version: Original Submission **Date:** 12/27/2017

Reviewer name: Martin Mascher

Reviewer Comments to Author:

The manuscript of Lu et al. describes the evaluation of Fossill libraries (Fosmid libraries adapted to Illumina paired-end sequencing) for validating and improving wheat genome assemblies. This technical paper is a timely contribution to the highly active research area of wheat genome assembly. A number of draft genome assembly of different wheat genotypes have been released in the past two years. The authors analyze all currently published assemblies and make their raw data available for future use. A very important finding is that the WGS strategies to chromosome-wise BAC-by-BAC sequencing. The manuscript is clearly written and easy to follow. The figures and tables are instructive. The manuscript can be published as is. I found only a few typos:

1 p 3, l. 60 avoids -> avoid
2 p. 15, l. 388 "Trimmomatic v0.32 (parameter)". Something is missing here, I think the parameters used for Trimmomatic.
3 p 15, l. 391 "was mapped" -> "were mapped"
4 p 16, l. 400/401 The abbreviation "sd" (standard deviation) has not been defined. Better spell out.
5 Legend of Table1: publically -> publicly
6 Num > n50 should be explained in a foot note.

Methods

Are the methods appropriate to the aims of the study, are they well described, and are necessary controls included? Yes

Conclusions

Are the conclusions adequately supported by the data shown? Yes

Reporting Standards

Does the manuscript adhere to the journal's guidelines on [minimum standards of reporting?]() Yes

Choose an item.

Statistics

Are you able to assess all statistics in the manuscript, including the appropriateness of statistical tests used? Yes, and I have assessed the statistics in my report.

Quality of Written English

Please indicate the quality of language in the manuscript: Acceptable

Declaration of Competing Interests

Please complete a declaration of competing interests, considering the following questions:

- Have you in the past five years received reimbursements, fees, funding, or salary from an organisation that may in any way gain or lose financially from the publication of this manuscript, either now or in the future?
- Do you hold any stocks or shares in an organisation that may in any way gain or lose financially from the publication of this manuscript, either now or in the future?
- Do you hold or are you currently applying for any patents relating to the content of the manuscript?
- Have you received reimbursements, fees, funding, or salary from an organization that holds or has applied for patents relating to the content of the manuscript?
- Do you have any other financial competing interests?
- Do you have any non-financial competing interests in relation to this paper?

If you can answer no to all of the above, write 'I declare that I have no competing interests' below. If your reply is yes to any, please give details below.

I declare that I have no competing interests.

I agree to the open peer review policy of the journal. I understand that my name will be included on my report to the authors and, if the manuscript is accepted for publication, my named report including any attachments I upload will be posted on the website along with the authors' responses. I agree for my report to be made available under an Open Access Creative Commons CC-BY license (http://creativecommons.org/licenses/by/4.0/). I understand that any comments which I do not wish to be included in my named report can be included as confidential comments to the editors, which will not be published.

I agree to the open peer review policy of the journal

To further support our reviewers, we have joined with Publons, where you can gain additional credit to further highlight your hard work (see: https://publons.com/journal/530/gigascience). On publication of this paper, your review will be automatically added to Publons, you can then choose whether or not to claim your Publons credit. I understand this statement.

Yes Choose an item.
