# Supplementary material for: Independent assessment and improvement of wheat genome sequence assemblies using Fosill jumping libraries
Source: Gigascience. 2018 May 11;7(5):giy053. doi: 10.1093/gigascience/giy053 (PMC5967450; doi:10.1093/gigascience/giy053)
Supplement: Reviewer_2_Report_(Original_Submission) -- Sachiko Isobe — 2/12/2018 Reviewed [file giy053_reviewer_2_report_(original_submission).pdf]

Reviewer Report

Title: Independent assessment and improvement of wheat genome sequence assemblies using Fosill jumping libraries.

Version: Original Submission **Date:** 2/12/2018

Reviewer name: Sachiko Isobe

Reviewer Comments to Author:

The manuscript assessed published wheat genome sequences assembled by different methodologies based on Ilumina reads of Fosill jumping libraries. Re-assembling with the Fosill jumping libraries were also performed, and suggested its effectivity. The results of comparison of different wheat assemblies and revealing the features of errors are interesting, and provides readers useful information especially for genome assembly in species with large genome and/or polyploidy. With these reasons, I concluded its acceptance with minor revision.

Minor comments
Fosill jumping library is a key technology in this study, however, there is no description about the library in background. Please add a brief explanation,

There are several description about long reads, especially SMRT in background and discussion. However, reads derived from SMRT did not used in this study. It is worth to mention about the technology, but no clear relation was described between SMRT and Fosill jumping library. Can the Fosill jumping library exist together with PacBio/Nanopore sequences? Or will it be taken place? I'd like to know the authors idea.

L90-95:
This section describes about the effectivity of long reads. However, long reads were not used in wheat assembly, and the description of L90-95 is not fit the section. Please reconsider the structure of the section.

L138 0.75x(>50x physical coverage) How did the author calculate 50x physical coverage?

Table 1 Percentages of consistent scaffolds and consistent bases, all numbers should be represent with first decimal place. Here only Denovo MAGIC represents with first decimal place.

Additional file 1
Table 3 and Fig5.
The description of 0X is not adequate because it represents 'no data'. The number should be replaced for example 0.1X.

Methods

Are the methods appropriate to the aims of the study, are they well described, and are necessary controls included? Yes

Conclusions

Are the conclusions adequately supported by the data shown? Yes

Reporting Standards

Does the manuscript adhere to the journal's guidelines on minimum standards of reporting? Yes

Choose an item.

Statistics

Are you able to assess all statistics in the manuscript, including the appropriateness of statistical tests used? No, and I do not feel adequately qualified to assess the statistics.

Quality of Written English

Please indicate the quality of language in the manuscript: Acceptable

Declaration of Competing Interests

Please complete a declaration of competing interests, considering the following questions:

- Have you in the past five years received reimbursements, fees, funding, or salary from an organisation that may in any way gain or lose financially from the publication of this manuscript, either now or in the future?
- Do you hold any stocks or shares in an organisation that may in any way gain or lose financially from the publication of this manuscript, either now or in the future?
- Do you hold or are you currently applying for any patents relating to the content of the manuscript?
- Have you received reimbursements, fees, funding, or salary from an organization that holds or has applied for patents relating to the content of the manuscript?
- Do you have any other financial competing interests?
- Do you have any non-financial competing interests in relation to this paper?

If you can answer no to all of the above, write 'I declare that I have no competing interests' below. If your reply is yes to any, please give details below.

I declare that I have no competing interests

I agree to the open peer review policy of the journal. I understand that my name will be included on my report to the authors and, if the manuscript is accepted for publication, my named report including any attachments I upload will be posted on the website along with the authors' responses. I agree for my report to be made available under an Open Access Creative Commons CC-BY license (http://creativecommons.org/licenses/by/4.0/). I understand that any comments which I do not wish to be included in my named report can be included as confidential comments to the editors, which will not be published.

I agree to the open peer review policy of the journal

To further support our reviewers, we have joined with Publons, where you can gain additional credit to further highlight your hard work (see: https://publons.com/journal/530/gigascience). On publication of this paper, your review will be automatically added to Publons, you can then choose whether or not to claim your Publons credit. I understand this statement.

Yes Choose an item.
